# Carotenoid Composition of *Telekia speciosa*

**DOI:** 10.3390/plants12244116

**Published:** 2023-12-08

**Authors:** Erzsébet Varga, Viktória Lilla Balázs, Viktor Sándor, Attila Agócs, Veronika Nagy, Sándor Balázs Király, Tibor Kurtán, Péter Molnár, József Deli

**Affiliations:** 1 Department of Pharmacognosy and Phytotherapy, George Emil Palade University of Medicine, Pharmacy, Science and Technology of Targu Mures, 540139 Târgu Mureș, Romania; 2 Department of Pharmacognosy, Faculty of Pharmacy, University of Pécs, Rókus utca 2, H-7624 Pécs, Hungary; 3 Institute of Bioanalysis, Medical School, University of Pécs, Szigeti út 12, H-7624 Pécs, Hungary; 4 Department of Biochemistry and Medical Chemistry, Medical School, University of Pécs, Szigeti út 12, H-7624 Pécs, Hungaryvera.nagy@aok.pte.hu (V.N.); 5 Department of Organic Chemistry, Faculty of Sciences, University of Debrecen, H-4032 Debrecen, Hungary

**Keywords:** *Teleki speciosa*, carotenoids, HPLC-DAD-MS analysis, NMR

## Abstract

The carotenoid composition of the flower of *Telekia speciosa* was investigated for the first time by HPLC-DAD-MS. In addition to the main carotenoid lutein and its geometrical isomers, 5,6-epoxy-carotenoids, namely violaxanthin, lutein 5,6-epoxide and antheraxanthin, were detected in larger amounts. In addition, β-carotene 5,6-epoxide and β-carotene 5,6,5′,6′-diepoxide were found, which occurs very rarely in plants. For unambigous identification, β-carotene 5,6-epoxide and β-carotene 5,6,5′,6′-diepoxide were prepared semisynthetically, and they were characterized by ^1^H and ^13^C NMR and HPLC-CD methods.

## 1. Introduction

*Telekia speciosa* (Schreb.) Baumg. (basionym–*Buphtalmum speciosum* Schreb.) is the only species classified in the genus Telekia Baumg. (*Asteraceae*). It can mostly be found in Southeastern Europe and Asia Minor. It is the only species belonging to the genus *Telekia* Baumg. and, based on recent studies [1], is related to the resiniferous taxa of *Inula* L., including *I. helenium*.

To date, little phytochemical work has been performed on *T. speciosa*, known as “yellow oxeye” in English [1,2,3,4]. Its leaves are triangular, doubly serrated, long and petiolated, and the flowers are yellow [5]. The roots of *T. speciosa* contain essential oils with eudesmane-type sesquiterpene lactones as major constituents [6]. The essential oils of different parts of *Telekia speciosa* contain more than 100 compound, which were determined by GC-MS-FID technique [7]. From the flowers, ferulic acid and caffeic acid derivatives were isolated: 6-*O*-(*E*)-caffeoyl-glucopyranose, and 3-*O*-caffeoylquinic acid (chlorogenic acid). An analysis showed the presence of a flavonol glucoside (patulitrin) as well [8]. However, the carotenoid composition of the *Telekia speciosa* flower has not been studied so far.

Carotenoids are present in different plant organs and show significant variability [9]. The flower petals of most plants accumulate both β,β- and β,ε-carotenoids. In flower petals, yellow xanthophylls can mainly be detected, such as lutein (**1**), β-cryptoxanthin (**2**), and zeaxanthin (**3**) (Figure S1). Carotenoid epoxides such as violaxanthin (**4**), antheraxanthin (**5**), neoxanthin (**6**), and lutein-5,6-epoxide (**7**) (Figure S1) are also common. Some flowers contain carotenes such as lycopene and β-carotene, as well as γ- and δ-carotene, and have a deep yellow to orange colour. 5,8-Epoxy carotenoids impart pale yellow and other xanthophylls impart deep yellow to orange colours to flowers, depending on the carotenoid content in the petals. Certain flowers show distinctive carotenoid profiles [10].

**Figure 1:**
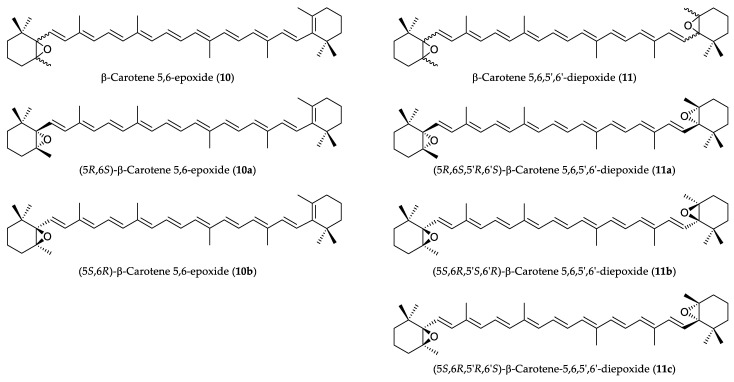
Structure of diastereomers of β-carotene-5,6-epoxides and 5,6,5′,6′-diepoxides.

Epoxy-carotenoids are important dietary carotenoids in many fruits and vegetables. Lutein (**1**), zeaxanthin (**3**) and some of their 5,6-epoxy derivatives (lutein 5,6-epoxide (**7**), antheraxanthin (**5**) and violaxanthin (**4**)) occur widely in nature [11]. However, carotenoids with 5,6-epoxy groups on a non-hydroxylated β-ring have rarely been reported. In the 1970s and 1980s, β-carotene 5,6-epoxide (**10**) *(*Figure 1) was isolated as a minor carotenoid from the algae of Prymnesiophycae [12], fruit of tamarillo (Cyphomandra betacea) [13], and the petals of some species of Medicago [14]. It was also detected in red mamey (Pouteria sapota) [15], but could not be isolated in its pure crystalline state. β-Carotene 5,6,5′,6′-diepoxide was only isolated from the tubers of the white-fleshed variety of sweet potato [16]. The total synthesis and characterization of (5*S*,6*R*)-β-carotene-5,6-epoxide (**10b**) and (5*R*,6*S*,5′*R*,6′*S*)-5,6,5′,6′-diepoxide (**11a**) were carried out in Eugster’s laboratory about 40 years ago [17,18].

As a continuation of our work on the carotenoid analysis of medicinal plants, we decided to investigate the carotenoid composition of *Telekia speciosa* inflorescences. Here, we report the results of a detailed HPLC-DAD-MS investigation of the full inflorences, petals and floret of *Telekia speciosa*. In addition, we describe the semisynthesis and characterization of β-carotene 5,6-epoxide (**10**) and β-carotene 5,6,5′,6′-diepoxide (**11**).

## 2. Results

### 2.1. Semisynthesis and Identification of β-Carotene-5,6-epoxide (***10***) and 5,6,5′,6′-Diepoxide (***11***)

Since the natural 5,6-epoxide (**10**) and 5,6,5′,6′-diepoxide (**11**) of β-carotene (**8**) were found to be very minor carotenoids, no conclusive structural elucidation could be carried out from the natural source. For the identification of minor carotenoids, at least three criteria must be fulfilled [19]: (a) the UV-Vis spectrum should be the same, (b) HPLC co-chromatography with an authentic sample is needed and (c) a proper mass spectrum is required.

To aid in the identification of β-carotene-5,6-epoxide (**10**) and 5,6,5′,6′-diepoxide (**11**), the compounds were prepared semisynthetically from β-carotene by epoxidation with monoperoxophthalic acid. The epoxidation reaction was carried out in our laboratory by Péter Molnár in the 1970s. The separation and purification of β-carotene mono- (**10**) and diepoxide (**11**) was achieved by open-column chromatography on Ca(OH)_2_ adsorbent. A detailed NMR analysis of the isolated products was undertaken in the 1990s at the Department of Organic Chemistry of the University of Bern, by Andrea Steck. The purity of the materials used for our study, produced nearly 50 years ago, was checked with HPLC and proton and carbon-13 NMR tests. The results (purity >95%, HPLC) show that carotenoids stored under the right conditions (crystalline, in an ampoule under N_2_ or Ar atmosphere, at −20 °C) do not decompose for a long time.

Carotenoids containing β-rings with or without substituents in the 3,3′-positions can be oxidized with perbenzoic acid or mono-peroxyphthalic acid. The epoxidation produced two diastereomeric 5,6-epoxides with (5*R*,6*S*) and (5*S*,6*R*) absolute configurations (Figure 1) [20]. The separation of *anti*-(3*S*,5*R*,6*S*) and *syn*-(3*S*,5*S*,6*R*) diastereomers of 3-hydroxy-5,6-epoxy carotenoids (β-cryptoxanthin-5,6-epoxide, capsanthin-5,6-epoxide) was and could be achieved by open-column chromatography or HPLC using C_18_ or C_30_ stationary phases [21]. The epoxidation of the nonhydroxylated β-ring also produced two diastereomeric 5,6-epoxides with (5*R*,6*S*) and (5*S*,6*R*) absolute configurations [22,23]. Usually, an unsubstituted β-ring makes it very difficult or impossible to separate such stereoisomers. Moreover, in the case of β-carotene diepoxide, three stereoisomers, (5*R*,6*S*,5′*R*,6′*S*)-(**11a**), (5*S*,6*R*,5′*S*,6′*R*)-(**11b**) and *meso*-(5*R*,6*S*,5′*S*,6′*R*)-(**11c**) isomers, are formed (Figure 1). In both of our cases, the separation of the diastereomers could only be accomplished by chiral HPLC column at an analytical scale.

For identification, 1D and 2D NMR analysis were performed for the diastereomeric mixtures of β-carotene-5,6-epoxides and β-carotene-5,6,5′,6′-diepoxides, respectively. The ^1^H and ^13^C signals were assigned by means of ^1^H and ^13^C, H,H-COSY, DEPT-135 and inverse-HMQC spectra. The proton chemical shifts in the epoxidated end groups (H-7 at δ 5.88 ppm; H-8 at δ 6.29 ppm) and the ^3^*J*_H,H_ coupling constants (*J*_7,8_ = 15.6 Hz) were in accordance with the corresponding data obtained from the literature [23,24]. The (5*R*,6*S*)- and (5*S*,6*R*)-5,6-epoxides of β-carotene are nondistinguishable by nuclear magnetic resonance [22,23].

**Figure 2:**
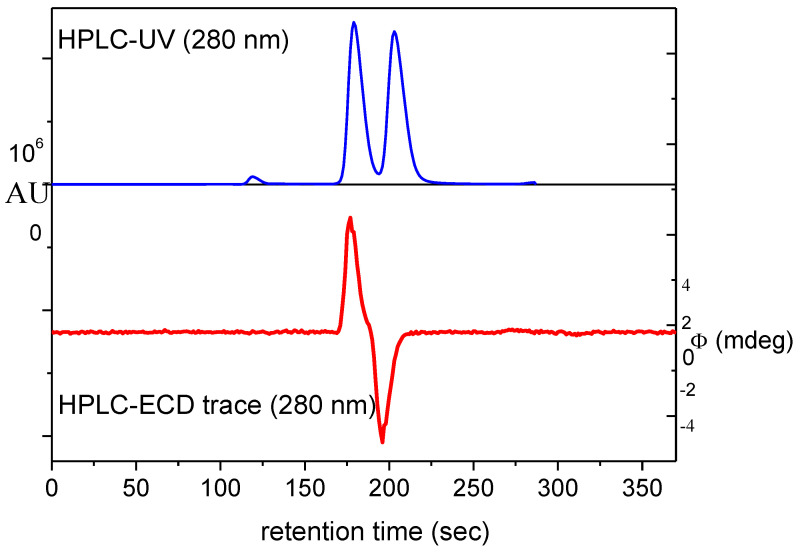
HPLC-UV (blue) and HPLC-ECD (red) chromatograms of the diastereomeric mixture of β-carotene-5,6-epoxides (5*R*,6*S*)-**10a** and (5*S*,6*R*)-**10b** monitored at 270 nm.

As previously mentioned, the epoxidation of β-carotene with monoperoxyphthalic acid was not stereoselective, since the two diastereomeric mono-epoxides formed in equal amounts (Figure 2). The separation of diastereomeric β-carotene-5,6-epoxides (5*R*,6*S*)-**10a** and (5*S*,6*R*)-**10b** was achieved on a Chiralpak IA column using hexane/dichloromethane 85:15 as an eluent.

**Figure 3:**
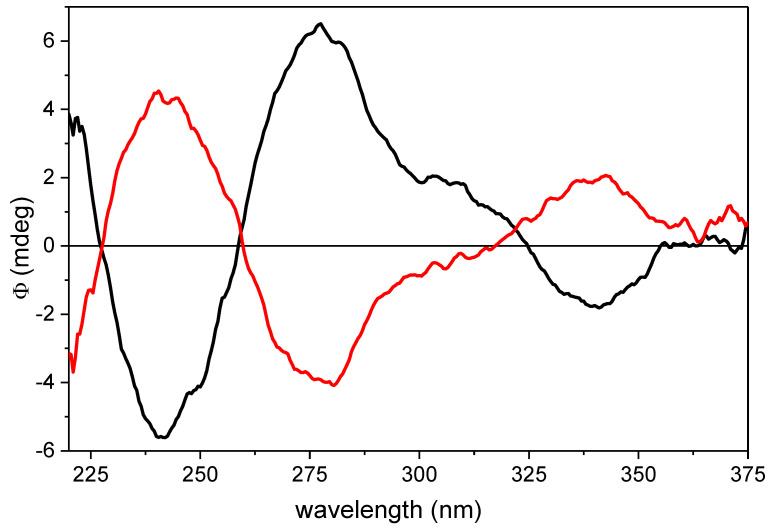
Online HPLC-ECD spectra of β-carotene-5,6-epoxide diastereomers (5*R*,6*S*)-(**10a**) (red curve, second eluting diastereomer), and (5*S*,6*R*)-(**10b**) (black curve, first eluting diastereomer), recorded in hexane/dichloromethane 85:15.

Since online HPLC-UV and HPLC-ECD measurements had been used efficiently for the investigation stereoisomeric mixtures of natural products [25,26,27], this technique was utilized in the separation of diastereomers. The HPLC-ECD chromatogram recorded at 270 nm showed opposite Cotton effects (CEs) for the two diastereomers. Online HPLC-ECD spectra were recorded by stopping the flow of the eluent in the HPLC-ECD flow cell at the maximum concentration of the separated diastereomers. The diastereomers had near-mirror-image ECD curves above 220 nm, allowing for the configurational assignment of the synthetic diastereomers (Figure 3). The CEs of natural*,* and semisynthetic β-carotene-5,6-epoxides had opposite signs above 240 nm, reflecting the different configuration of the 5,6-epoxy end groups. The first eluting diastereomer was the semisynthetic (5*S*,6*R*) stereoisomer, while the second eluting diastereomer was the natural one (5*S*,6*R*). These data corroborated the reported values of (5′*S*,6′*R*)- and (5′*R*,6′*S*)-β-cryptoxanthin-5′,6′-epoxide [23].

**Figure 4:**
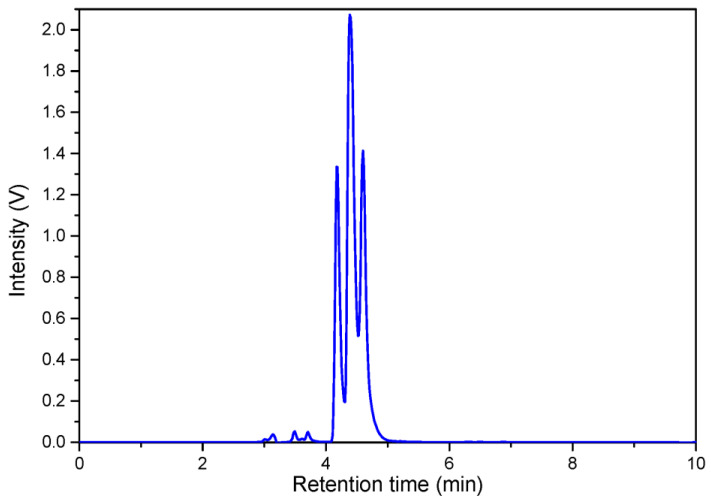
HPLC-UV and HPLC-CD chromatograms of a mixture of (5*S*,6*R*,5′*S*,6′*R*)-(**11a**) (peak 1) (5*R*,6*S*,5′*S*,6′*R*)-(**11c**) (peak 2) and (5*R*,6*S*,5′*R*,6′*S*)-(**11b**) (peak 3) β-carotene-5,6,5′,6′-depoxide diastereomers.

**Figure 5:**
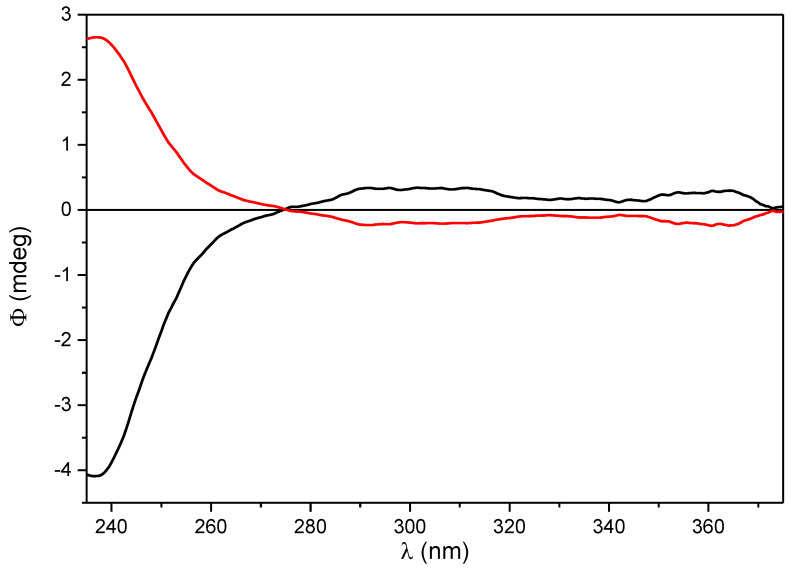
Online HPLC-ECD spectra of stereoisomeric β-carotene-5,6,5′,6′-diepoxides (5*S*,6*R*,5′*S*,6′*R*)-**11a** (black curve, first-eluting stereoisomer) and (5*R*,6*S*,5′*R*,6′*S*)-**11b** (red curve, third-eluting stereoisomer) recorded in hexane/dichloromethane 75:25.

The separation of diastereoisomers of β-carotene diepoxide (**11**) was successful on the Chiralpak IA-3 column using hexane/dichloromethane 75:25 as an eluent (Figure 4). Even though baseline separation could not be achieved, three peaks were observed and weak ECD spectra were recorded for the enantiomeric diepoxides, the first- and third-eluting peaks. Due to the weak ECD signals, enantiomeric correction was used instead of correction with blank solvent as the background. Near-mirror-image ECD spectra were obtained after smoothing, and the assignment of the absolute configuration of the enantiomers was performed on the basis of our previous results [22,23] using the sign of the CE below 240 nm (Figure 5). The first-eluting peak was identified as the (5*S*,6*R*,5′*S*,6′*R*)-diepoxide, while the third-eluting peak belonged to the (5*R*,6*S*,5′*R*,6′*S*)-diepoxide. The second-eluting stereoisomer, with roughly twice the UV intensity of the other two and a baseline ECD curve, was identified as the *meso* compound.

### 2.2. Analysis of Telekia speciosa

The HPLC-DAD and HPLC-DAD-MS analyses were performed using a C_30_ phase. The carotenoids were identified by their elution order on the C_30_ HPLC column via spiking with authentic standards, UV-visible spectra (λ_max_, spectral fine structure (%III/II)), *cis* peak intensity (%A_B_/A_II_) and mass spectrum compared to standards and the data available in the literature [19,21].

**Figure 6:**
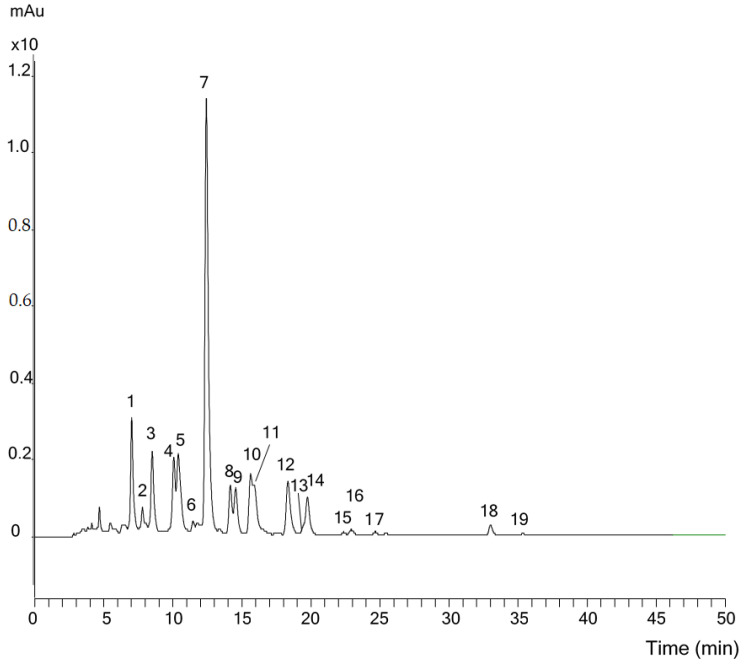
HPLC-DAD full chromatogram of *Teleki speciosa* petals. Peak numbering is given in Table 1.

In all the investigated *T. speciosa* extracts, 19 compounds were detected by HPLC at a 450 nm wavelength (Figure 6 and Table 1). The main component, Peak 7, was identified as (all-*E*)-lutein (**1**) by its UV-VIS (443, 470 nm) and MS spectra (*m*/*z* 551, [M + H − H_2_O]^+^). Peaks 10 and 12 were attributed to (9*Z*)- and (9′*Z*)-isomers of lutein (**1**), according to the characteristic hypsochromic shift, the intensity of the *cis* peak in the UV-visible spectra and the MS ([M + H − H_2_O]^+^ at *m*/*z* 551), and co-chromatography with the iodine-catalyzed isomerization mixture of lutein (**1**).

Peak 6 was found to be (13′*Z*)-lutein because of the hypsochromic shift of λ_max_, and the high intensity of the *cis* peak in the UV spectrum. Its *m*/*z* value was 551, co-chromatography with the iodine-catalyzed isomerization mixture of lutein (**1**) confirming to the (13′*Z*)-isomer. The other *cis* isomer of lutein (**1**), (13*Z*)-lutein was covered by peak 5, and could be identified by EIC spectra at *m*/*z* 551.

(All-*E*)-violaxanthin (**4**, peak 1) and (9*Z*)-violaxanthin ((9*Z*)-**4**, peak 5) were identified by UV-visible spectra and by the hypsochromic shift of 4 nm for peak 5 to the all-*E* isomer. Co-elution with standards and their *m*/*z* value ([M + H]^+^ 601) corroborated the identification.

From the polar carotenoids, (9*Z*)-neoxanthin ((9*Z*)-**6**) (Peak 2) showed a characteristic UV-visible spectra (λ_max_ 411, 434, 463 nm) with a fine structure. The identity of peak 2 was supported by co-elution with an authentic standard and its *m*/*z* value (601).

The next major component, Peak 3, provided λ_max_ at 415, 438, 467 nm in UV with a fine structure and a 567 *m*/*z* value ([M + H − H_2_O]^+^) in the MS spectra. After spiking with a standard isolated from the petals of *Chelidonium majus*, it was confirmed to be all-*trans*-lutein 5,6-epoxide (**7**).

Peak 4 had a UV-visible spectra similar to that of lutein (**1**). The molecular masses, detected at 584 seemed to correspond to (all-*E*)-antheraxanthin (**5**). This assumption was confirmed by co-elution with the authentic standard.

Peaks 14 and 16 provided a UV-visible spectrum similar to that of peak 7. The molecular masses ([M + H]^+^), detected at *m*/*z* 553 for both compounds, seemed to correspond to (all-*E*)-α-cryptoxanthin (**9**), and (all-*E*)-β-carotene 5,6-epoxide (**10**). These assumptions were confirmed by spiking with the authentic standards.

Peak 8 had a UV-visible spectra similar to that of peak 1 and 3 (λ_max_: 418, 437, 468 nm, (%III/II: 91)). The molecular mass ([M + H]^+^), detected at *m*/*z* 569 for the compound, showed that the molecule contained two oxygen atoms. Based on the fine structure of the UV-visible spectrum and the retention time of this peak, it was identified as β-carotene-5,6,5′,6′-diepoxide (**11**). These were confirmed by co-chromatography with the authentic standards produced in our laboratory.

In plants, lutein (1) is usually accompanied by an approximately equal amount of zeaxanthin (3). Peak 9 was identified as zeaxanthin (**3**) by its UV-vis (λ_max_: 450, 476 nm, %III/II: 22) and MS spectra (*m*/*z* 569, ([M + H]^+^).

Peak 18 had a UV-visible spectra similar to that of zeaxanthin (**3**). The *m*/*z* value of 537 suggested that this was (all-*trans*)-β-carotene (**8**). Peak 19 was the 9*Z*-isomer of β-carotene (**8**) based on its UV-VIS and MS spectra.

## 3. Discussion

Flowers were collected at two different places, but no significant differences were observed in the total carotenoid content. The petals contained slightly more carotenoids, while the carotenoid content of the floret did not even reach a tenth of the inflorescences.

One part of the work focused on the identification of carotenoids in fresh flowers of *T. speciosa* using HPLC. Similarly to some other flowers, lutein and its geometrical isomers were the major carotenoids. The amounts of (all-*E*)-lutein varied between 38% and 44% in inflorences, florets and petals. The proportion of (9*Z*)- and (9′*Z*)-lutein isomers was almost the same, except for floret, where the amount of the (9′*Z*)-isomer reached 11%, whereas in petals it was only approx. 1%. Other hydroxy carotenoids are represented by zeaxanthin (**3**) and α-cryptoxanthin (**9**), as well as their *cis*-isomers. Epoxy-carotenoids, namely violaxanthin (**4**), lutein 5,6-epoxide (**7**) and antheraxanthin (**5**), were found in higher amounts (5–8%) in the petals and in the whole inflorescence. The co-occurrence of these three carotenoids in flowers is quite rare. Usually, besides violaxanthin (**4**), some neoxanthin can mainly be detected. In some cases, violaxanthin (4) can be found, in addition to larger amounts of lutein epoxide (**7**), but these flowers (*Inula helenium*, *Cytisus nigricans*) do not contain lutein (**1**) [unpublished]. The floret did not contain the 5,6-epoxides, but contained their furanoids. Due to the complexity of the system, we could not clearly identify these peaks. To our surprise, we found a larger amount of β-carotene diepoxide (**11**) and a smaller amount of β-carotene monoepoxide (**10**). In petals, the amount of β-carotene diepoxide enriched to 10%, while in the tubular flower it could not detected.

The occurrence of the 5,6-epoxy and 5,6,5′,6′-diepoxy β-carotene, besides the violaxanthin and lutein 5,6-epoxide, is rather rare in nature. The detection of these compounds indicates that not only β-carotene hydroxylase, but also the enzyme β-carotene epoxidase, works in the Telekia speciosa flower. In plants, generally, β-carotene hydroxylase produces zeaxanthin, which is further converted into antheraxanthin and violaxanthin by the enzyme zeaxanthin epoxidase. The hydroxylation of β-carotene usually takes place faster than epoxidation, so no β-carotene epoxides can be detected.

In order to clearly identify the occurrence of β-carotene 5,6-epoxide (**10**) and 5,6,5′,6′-diepoxide (**11**), they were prepared semisynthetically from β-carotene (**8**) and their structure was confirmed by NMR studies. This is the first report of the complete ^1^H and ^13^C NMR data of these carotenoids. The resulting stereoisomers were successfully separated during a chiral stationary phase; the configuration assigned to the peaks was performed using the on-line HPLC-ECD method.

## 4. Materials and Methods

### 4.1. Plant Materials, Pigment Extraction, Determination of Carotenoid Content

The flowers of *Telekia speciosa* were collected from Szováta and Bálványosfürdő (Maros country, Transylvania, Romania) in summer, 2019. The wild plant material was collected and identified by Erzsébet Varga, and example vouchers were deposited in the Department of Pharmacognosy and Phytotherapy, George Emil Palade University of Medicine, Pharmacy, Science and Technology of Targu Mures. The voucher specimen number was FS/0107/2019.

Analytical-grade chemicals were used for the extractions. The fresh plant samples (20–40 g) were extracted twice with acetone and once with Et_2_O. After evaporation, the residue of the acetonic extracts was dissolved in Et_2_O. The ethereal solutions were combined, and this total extract was saponified in heterogeneous phase (30% KOH/MeOH) overnight. The reaction mixture was washed with water 10 times. The saponified pigments were stored in benzene at −20 °C under nitrogen. The HPLC sample was prepared immediately before measurement. The benzene solution was evaporated to dryness and dissolved in *tert*-butyl methyl ether (MTBE)–methanol (MeOH) mixture.

The total carotenoid content was estimated spectrophotometrically in benzene at 450 nm (E_1%1cm_ = 2300) using a Jasco V-530 Spectrophotometer (JASCO Corporation 2967-5 Ishikawamachi Hachioji-shi, Tokyo, Japan) [19].

### 4.2. General Experimental Procedures

The UV-VIS spectra were recorded with a Jasco V-530 spectrophotometer in benzene.

NMR spectra were recorded with a Bruker DRX4OO (^1^H: 400.14 MHz; ^13^C: 100.61 MHz) and a Bruker Avance III Ascend 500 spectrometer (500/125 MHz for ^1^H/^13^C) in CDCl_3_. Chemical shifts are referenced as Me_4_Si (^1^H), or the residual solvent signals (^13^C). Solvents for the HPLC analysis (MeOH: methanol, MTBE: *tert*-butyl methyl ether, acetone) were of HPLC grade.

#### 4.2.1. Equipment for HPLC-DAD Separations on a C_30_ Stationary Phase

The HPLC analysis was performed by Dionex 3000 HPLC system (Thermo Fisher Scientific Inc., Waltham, MA, USA). Chromatograms were detected at 450 nm wavelength; data acquisition was performed by Chromeleon 7.20 software. The separation was carried out on an endcapped C_30_ column (250 × 4.6 mm i.d.; YMC C_30_, 3 µm, YMC Europe GmbH, Dinslaken, Germany). Eluents: (A) MeOH: MTBE (methyl-*t*-butylether): H_2_O = 81:15:4 *v*/*v*%; (B) MeOH: MTBE: H_2_O = 6:90:4 *v*/*v*%. The chromatography was performed in a linear gradient from 100% A eluent to 50% B mixture in 45 min, with 1.00 mL/min flow rate, at 22 °C.

#### 4.2.2. Equipment for HPLC-MS Separations at a C_30_ Stationary Phase

An Agilent 6350 Accurate-Mass Q-TOF LC/MS apparatus was used; data acquisition was performed by Agilent MassHunter Qualitative Analysis B.04.00 (Agilent Technologies, Santa Clara, CA, USA). For LC-(APCI)-MS, the positive ion mode was used with TIC; scanning range 200–1500 *m*/*z*; corona voltage 2.6 kV; fragmentor voltage 150 V; skimmer 60 V; Oct 1RF Vpp 750 V. The flow rate of the dried nitrogen as nebulizer gas was 240 L/h, and the vaporizer temperature was 400 °C. Column: 250 × 4.6 mm i.d.; YMC C_30_, 3 μm. Eluents: (A) MeOH: MTBE: H_2_O = 81:15:4 *v*/*v*% (B) MeOH: MTBE: H_2_O = 6:90:4 *v*/*v*%. The chromatograms were performed in linear gradient from 100% A to 100% B eluent in 90 min at 22 °C. Flow rate: 1.00 mL/min.

#### 4.2.3. Equipments for Chiral HPLC and HPLC-ECD Analysis

Chiral HPLC separations were carried out with a Jasco HPLC system on Chiralpak IA column (0.46 cm × 25 cm, 5 μm) using *n*-hexane:dichloromethane = 85:15 at a flow rate of 1 mL/min for **5**, **7** and **10**, and Chiralpak IA column (0.46 cm × 25 cm, 3 μm) using *n*-hexane:dichloromethane = 75:25 for **11**. HPLC-UV chromatograms were measured with a Jasco MD-910 multiwavelength detector (JASCO Corporation, 2967-5 Ishikawamachi Hachioji-shi, Tokyo, Japan). The baseline of the chromatograms was reduced to zero immediately after the start of each run; this allowed for the measurement of the relative absorbance. The HPLC-ECD traces were recorded at the specified wavelength with a Jasco J-810 ECD spectropolarimeter (JASCO Corporation, 2967-5 Ishikawamachi Hachioji-shi, Tokyo, Japan) equipped with a 1 cm path length HPLC flow cell, and the baseline was reduced to zero after the start of each run. The online ECD and UV spectra were recorded simultaneously by stopping the flow at the UV absorption maximum of each peak. ECD ellipticity values (ϕ) were not corrected for concentration. For an HPLC-ECD spectrum, three consecutive scans were recorded and averaged with 2 nm bandwidth, 1 s response, and standard sensitivity. The HPLC-ECD spectrum of the eluent, recorded in the same way, was used as background. The concentration of the injected sample was set so that the HT (voltage) value did not exceed 500 V in the HT channel.

#### 4.2.4. Identification of the Peaks

The carotenoids were identified using the following data: elution order on the C_30_ HPLC column, spiking with authentic standards, UV-visible spectrum (λ_max_, spectral fine structure (% III/II), *cis* peak intensity (% AB/II)) and mass spectrum (molecular ion and fragments) compared to standards and the data available in the literature [21]. Authentic samples were taken from our collection.

### 4.3. Preparation of β-Carotene 5,6-Epoxide (**10**) and β-Carotene 5,6,5′,6′-Diepoxide (**11**)

A total of 10 mL of 0.25 M concentration monoperoxyphthalic acid (Et_2_O solution) was added to a solution of β-carotene (**8**) (400 mg) in Et_2_O (800 mL) at room temperature. The mixture was kept under N_2_, in the dark. After 21 h, the mixture was washed with 5% aq. NaHCO_3_ solution, the organic phase was dried (Na_2_SO_4_) and the solvent was evaporated.

The residue was dissolved in hexane and submitted to open-column chromatography (Ca(OH)_2_, eluent: hexane). Picture after development: 15 mm bright yellow (*Fraction 1*, furanoids); 30 mm intermediate zone; 40 mm bright yellow (*Fraction 2*, mixture of β-carotene-diepoxide (**11**)); 15 mm intermediate zone; 50 mm pale ochre (*Fraction 3*, β-carotene 5,6-epoxides (**10**)), 20 mm orange zone (*Fraction 4*, β-carotene (**8**)). After processing, *Fraction 2* and *Fraction 3* were crystallized from toluene through the addition of methanol yielding: 50 mg of a mixture of β-carotene-5,6,5′,6′-diepoxide and 102 mg of β-carotene 5,6-epoxide, respectively (**11**, **10**).

Mixture of (5*R*,6*S*)-(**10a**) and (5*S*,6*R*)-β-carotene-5,6-epoxide (**10b**): orange crystals, mp. 143 °C, UV-VIS (benzene): λ_max_ 434, 457, and 487 nm, λ_max_ after acid treatment: 416, 438, 465 nm; ^1^H NMR (400 MHz, CDCl_3_): δ 0.94 (3H, *s*, Me-17)*; 1.03 (6H, *s*, Me-16′, Me-17′); 1.07 (1 H, overlapping *dt*, H-2_a_); 1.10 (3H, *s*, Me-16)*; 1.15 (3H, *s*, Me-18); 1.40–1.43 (2H, *m*, H-3); 1.44–1.48 (2H, *m*, H-2′); 1.48 (1H, overlapping *dt*, H-2_b_); 1.60–1.63 (2H, *m*, H-3′); 1.72 (3H, *s*, H-18′); 1.73 (1H, overlaping *dt*, H-4_a_); 1.89 (1H, *dt*, *J*_4a/4b_ = 15.7 Hz, *J*_4b/3_ = 8.3 Hz, H-4_b_); 1.93 (3H, *s*, Me-19); 1.96 (3H, *s*, Me-20); 1.97 (6H, *s*, Me-19′, Me-20′); 2.02 (2H, *t*, *J*_3′,4′_ = 5.8 Hz, H-4′); 5.88 (1H, *d*, *J*_7,8_ = 15.6 Hz, H-7); 6.14 (1H, *d*, *J*_8′,7′_ = 15.6 Hz, H-8′); 6.15 (1H, overlaping *d*, *J*_10′/11′_ = 10.8 Hz, H-10′); 6.16 (1H, *d*, *J*_7′,8′_ = 15.6 Hz, H-7′); 6.19 (1H, *d*, *J*_10,11_ = 11.4 Hz, H-10,); 6.25–6.27 (2H, *m*, H-14, H-14′); 6.29 (1H, *d*, *J*_8,7_ = 15.6 Hz, H-8); 6.35 (1H, *d*, J_12′/11′_= 14.9, Hz H-12′); 6.36 (1H, *d*, *J*_12,11_ = 14.9 Hz, H-12); 6.60 (1H, *dd*, *J*_11/10_ = 11.4 Hz, *J*_11/12_ = 14.9 Hz, H-11); 6.62–6.64 (2H, *m*, H-15, H-15′); 6.66 (1H, *dd*, *J*_11′/10′_ = 10.8 Hz, *J*_11′/12′_=14.9 Hz, H-11′). ^13^C NMR (100 MHz, CDCl_3_): δ 12.76 (C-20′), 12.78 (C-20), 12.82 (C-19′); 12.98 (C-19), 17.10 (C-3); 19.25 (C-3′); 21.15 (C-18); 21.76 (C-18′); 25.88 (C-17)**; 25.98 (C-16)**; 28.97 (C-16′, C-17′), 30.09 (C-4); 33.10 (C-4′); 33.82 (C-1); 34.27 (C-1′); 35.73 (C-2); 39.64 (C-2′); 65.51 (C-5); 71.38 (C-6); 124.11 (C-7); 124.68 (C-11); 125.11 (C-11′), 126.68 (C-7′), 129.38 (C-5′), 129.87 (C-15′); 130.23 (C-15); 130.82 (C-10′); 131.94 (C-10); 132.35 (C-14′); 132.79 (C-14); 134.42 (C-9); 136.07 (C-9′); 136.25 (C-13); 136.64 (C-13′); 137.20 (C-12′); 137.23 (C-8); 137.75 (C-8′); 137.91 (C-6′); 137.98 (C-12). * and ** Assignment may be exchanged; axial and equatorial positions for H-2 and H-4 cannot be distinguished.

The isomers were separated using a Chiralpak IA column (0.46 × 150 mm, 5 μm) (*n*-hexane/dichloromethane 85:15) and online HPLC-ECD spectra were recorded.

**10a**: t_R_ = 2.93 min. HPLC-ECD [nm (Φ), *n*-hexane/dichloromethane 85:15]: 341 (−1.82), 303 (2.05), 277.5 (6.51), 242 (−5.61), 222 (3.76).

**10b**: t_R_ = 3.27 min. HPLC-ECD [nm (Φ), *n*-hexane/dichloromethane 85:15]: 341 (1.94), 303 (−0.50), 277.5 (−3.92), 242 (4.20), 221 (−3.69).

Mixture of (5*R*,6*S*,5′*R*,6ʹ*S*)-(**11a**), (5*S*,6*R*,5′*S*,6ʹ*R*)-(**11b**) and (5*R*,6*S*,5′*S*,6′*R*)-β-carotene-5,6,5′,6′-diepoxide (**11c**): orange crystals, mp. 182–184 °C, UV-VIS (benzene): λ_max_ 428, 453, and 483 nm, λ_max_ after acid treatment: 388, 410, 436 nm; ^1^H NMR (400 MHz, CDCl_3_): δ 0.94 (6H, *s*, Me-17, Me-17′)*; 1.03–1.08 (2H, *m*, H-2_a_, H-2_a_’); 1.10 (6H, *s*, Me-16, Me-16′)*; 1.14 (6H, *s*, Me-18, Me-18′); 1.37–1.41 (2H, *m*, H-3, H-3′); 1.48 (2H, *dt*, *J*_2b,2a_ = 14.0 Hz, *J*_2b,3_ = 7.2 Hz, H-2_b_, H-2_b_′); 1.74 (2H, *dt*, *J*_4a,4b_ = 15.3 Hz, *J*_4a,3_ = 5.7 Hz, H-4_a_, H-4_a_′); 1.91 (2H, *dt*, *J*_4a,4b_ = 15.3 Hz, *J*_4b,3_ = 8.1 Hz, H-4_b_, H-4_b_′); 1.93 (6H, *s*, Me-19, Me19′); 1.96 (6H, *s*, Me-20, Me20′); 5.88 (2H, *d*, *J*_7,8_ = 15.6 Hz, H-7, H-7′), 6.19 (2H, *d*, *J*_10,11_ = 11.5 Hz, H-10, H-10′,); 6.24–6.27 (2H, *m*, H-14, H-14′); 6.29 (2H, *d*, *J*_8,7_ = 15.6 Hz, H-8, H-8′); 6.36 (2H, *d*, *J*_12,11_ = 14.9 Hz, H-12, H-12′); 6.61 (2H, *dd*, *J*_11/10_ = 11.5 Hz, *J*_11/12_ = 14.9 Hz, H-11, H-11′); 6.62–6.64 (2H, *m*, H-15, H-15′); ^13^C NMR (100 MHz, CDCl_3_): δ 12.79 (C-20, C-20′); 12.99 (C-19, C19′); 17.10 (C-3, C3′); 21.15 (C-18, C18′); 25.88 (C17, C17′)**; 25.98 (C-16, C16′)**; 30.09 (C-4, C4′); 33.82 (C-1, C1′); 35.73 (C-2, C2′); 65.51 (C-5, C5′); 71.39 (C-6, C6′); 124.14 (C-7, C7′); 124.77 (C-11, C11′); 130.12 (C-15, C15′); 131.92 (C-10, C10′); 132.73 (C-14, C14′); 134.48 (C-9, C9′); 136.42 (C-13, C13′); 137.22 (C-8, C8′); 137.95 (C-12, C12′). * and ** Assignment may be exchanged; axial and equatorial positions for H-2 and H-4 cannot be distinguished.

The isomers were separated using a Chiralpak IA column (0.46 × 250 mm, 3 μm) (*n*-hexane/dichloromethane 75:25) and online HPLC-ECD spectra were recorded.

**11a**: t_R_ = 4.19 min. HPLC-ECD [nm (Φ), *n*-hexane/dichloromethane 75:25]: 364 (0.30), 301.5 (0.34), 236.5 (−4.09).

**11b**: t_R_ = 4.39 min. HPLC-ECD [nm (Φ), hexane/dichloromethane 75:25]: 364 (−0.24), 301.5 (−0.21), 237 (2.65).

**11c**: t_R_ = 4.60 min.

## Figures and Tables

**Table 1:** Carotenoid composition of *Telekia speciosa*.

No.	Peak Name	UV-VIS	MS	Szováta Inflorence	Bálványos Inflorence	Szováta Petals	Szováta Floret
		λ_max_ [nm]	*m*/*z*	% of Total Carotenoids *			
1	Violaxanthin	416, 439, 468	601 [M + H]^+^	6.07	5.93	6.21	0.83
2	(9*Z*)-Neoxanthin	413, 436, 464	601 [M + H]^+^	0.74	0.65	1.24	2.33
3	Lutein 5,6-epoxide	416, 439, 468	567 [M − H_2_O + H]^+^	4.90	6.92	6.10	
4	Antheraxanthin	444, 471	585 [M + H]^+^	5.56	7.01	8.43	
5	(9*Z*)-Violaxanthin + (13*Z*)-Lutein	415, 436, 463	601 [M + H]^+^; 551 [M − H_2_O + H]^+^	7.77	6.73	8.08	
6	(13′*Z*)-Lutein	331, 436, 464	551 [M − H_2_O + H]^+^	1.25	1.77	2.83	
7	Lutein	444, 472	551 [M − H_2_O + H]^+^	38.62	42.71	40.25	43.80
8	β-Carotene diepoxide	419, 441, 468	569 [M + H]^+^	3.78	3.61	10.20	0
9	Zeaxanthin	450, 476	569 [M + H]^+^	4.76	4.20	1.67	5.16
10	(9*Z*)-Lutein	330, 441, 466	551 [M − H_2_O + H]^+^	6.37	3.91	6.55	9.77
11	Aurochrome	384, 401, 425	569 [M + H]^+^	5.43	4.92	2.27	1.43
12	(9′*Z*)-Lutein	332, 440, 466	551 [M − H_2_O + H]^+^	6.83	5.25	0.92	11.63
13	(9*Z*)-Zeaxanthin	340, 444, 469	569 [M + H]^+^	0.79	0.59	1.14	1.41
14	α-Cryptoxanthin	445, 472	553 [M + H]^+^	4.32	2.54	2.35	5.68
15	(9*Z*)-α-Cryptoxanthin	331, 440, 467	553 [M + H]^+^	0.31	0.18	0.11	0.58
16	β-Carotene 5,6-epoxide	444, 471	553 [M + H]^+^	0.77	0.74	0.48	0.60
17	β-Carotene 5,8-epoxide	426, 451	553 [M + H]^+^	0.43	0.58	0.25	0.86
18	β-Carotene	450, 475	537 [M + H]^+^	1.19	1.49	0.76	1.03
19	(9*Z*)-β-Carotene	446, 469	537 [M + H]^+^	0.14	0.26	0.15	0.32
	Total carotenoid (mg/g)			0.211	0.370	0.421	0.018

* Percentage of peak area in the HPLC chromatogram at 450 nm.

## Data Availability

Data are contained within the article and Supplementary Materials.

## Supplementary Materials

The following supporting information can be downloaded at: https://www.mdpi.com/article/10.3390/plants12244116/s1, Figure S1. Structure of carotenoids, Figures S2–S5. ^1^H-, ^13^C-, ^1^H,^1^H-COSY, and ^1^H,^13^C-HMQC NMR spectra of β-carotene 5,6-epoxide (**10**), Figures S6–S9. ^1^H-, ^13^C-, ^1^H,^1^H-COSY, and ^1^H,^13^C-HMQC NMR spectra of β-carotene 5,6,5′,6′-diepoxide (**11**), Figure S10. UV-vis and EIC chromatogram of *Teleki speciosa* flower extract, Figure S11. UV-vis spectra of catotenoids in *Teleki speciosa* flower extract detected by HPLC-DAD.
