# Supplementary material for: Carotenoid Composition of Telekia speciosa
Source: Plants (Basel). 2023 Dec 8;12(24):4116. doi: 10.3390/plants12244116 (PMC10747434; doi:10.3390/plants12244116)

Supplementary materials

Carotenoid composition of *Telekia speciosa*

Erzsébet Varga 1, Viktória Lilla Balázs 2, Viktor Sándor 3, Attila Agócs 4, Veronika Nagy 4, Sándor Balázs Király 5, Tibor Kurtán 5, Péter Molnár 2 and József Deli 2,4,*

[1] Department of Pharmacognosy and Phytotherapy, George Emil Palade University of Medicine, Pharmacy, Science and Technology of Targu Mures, 540139 Târgu Mureș, Romania
[2] Department of Pharmacognosy, Faculty of Pharmacy, University of Pécs, Rókus utca 2, H-7624 Pécs, Hungary
[3] Institute of Bioanalysis, Medical School, University of Pécs, Szigeti út 12, H-7624 Pécs, Hungary
[4] Department of Biochemistry and Medical Chemistry, Medical School, University of Pécs, Szigeti út 12, H-7624 Pécs, Hungary
[5] Department of Organic Chemistry, Faculty of Sciences, University of Debrecen, H-4032 Debrecen, Hungary
* Correspondence: jozsef.deli@aok.pte.hu

Figure S1. Structure of carotenoids

Figure S2-5. ^{1}H-, ^{13}C-, ^{1}H,^{1}H-COSY, and ^{1}H,^{13}C-HMQC NMR spectra of β–carotene 5,6-epoxide (**10**)

Figure S6-9. ^{1}H-, ^{13}C-, ^{1}H,^{1}H-COSY, and ^{1}H,^{13}C-HMQC NMR spectra of β-carotene 5,6,5′,6′-diepoxide (**11**)

Figure S10. UV-vis and EIC chromatogram of *Teleki speciosa* flower extract

Figure S11. UV-vis spectra of carotenoids in *Teleki speciosa* flower extract detected by HPLC-DAD

Figure S1. Structure of carotenoids

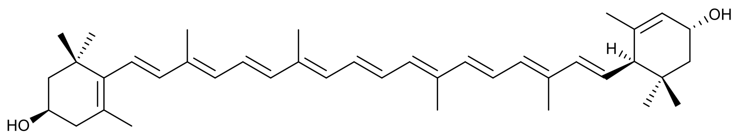

Lutein (**1**)

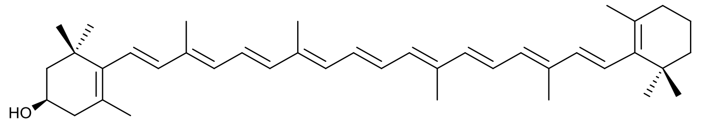

β-Cryptoxanthin (**2**)

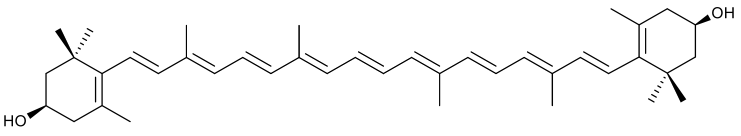

Zeaxanthin (**3**)

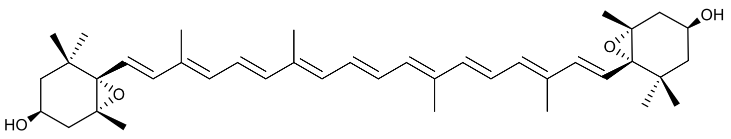

Violaxanthin (**4**)

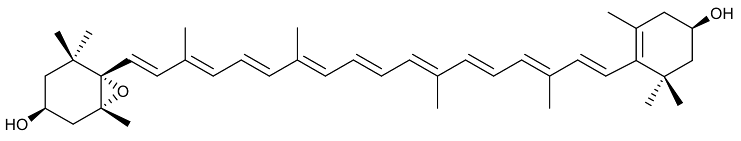

Antheraxanthin (**5**)

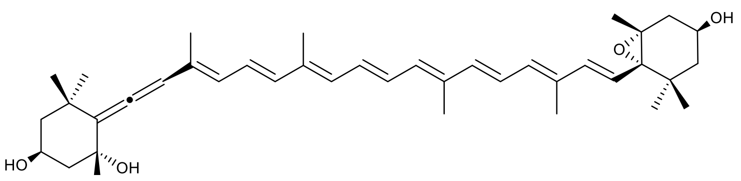

(all-*E*)-Neoxanthin (**6**)

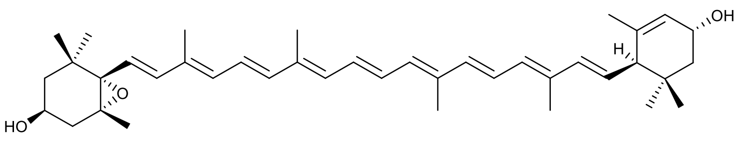

Lutein 5,6-epoxide (**7**)

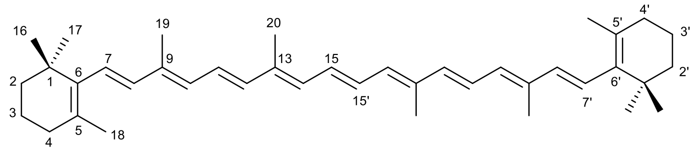

β-Carotene (**8**)

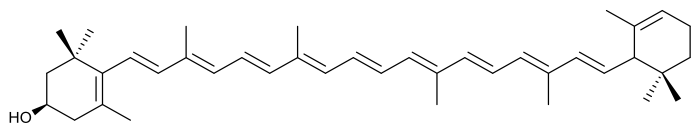

α-Cryptoxanthin (**9**)

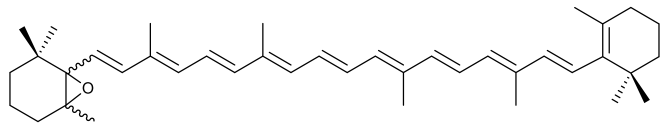

β-Carotene 5,6-epoxide (**10**)

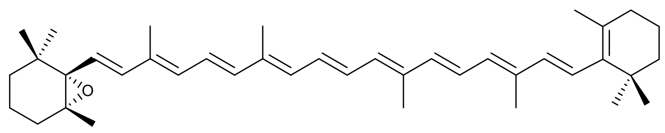

(5*R*,6*S*)-β-Carotene 5,6-epoxide (**10a**)

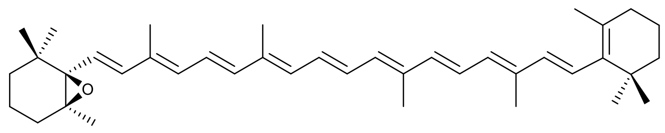

(5S,6*R*)-β-Carotene 5,6-epoxide (**10b)**

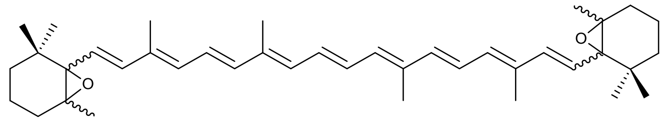

β-Carotene 5,6,5′,6′-diepoxide (**11**)

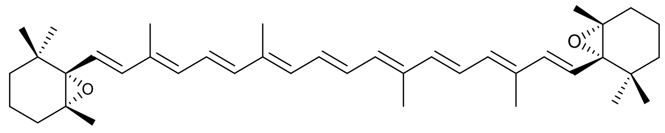

(5*R*,6*S*,5′*R*,6′*S*)-β-Carotene 5,6,5′,6′-diepoxide (**11a**)

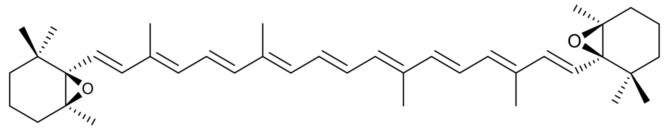

(5*S*,6*R*,5′*S*,6′*R*)-β-Carotene 5,6,5′,6′-diepoxide (**11b**)

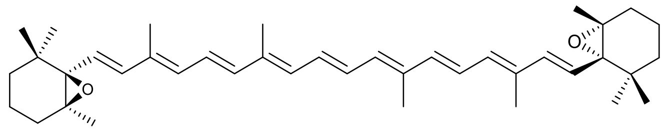

(5*S*,6*R*,5′*R*,6′*S*)-β-Carotene-5,6,5′,6′-diepoxide (**11c**)

Figure S2.a. ^{1}H-NMR spectrum of β-carotene 5,6-epoxide (**10**) in $CDCl_3$ (400 MHz)

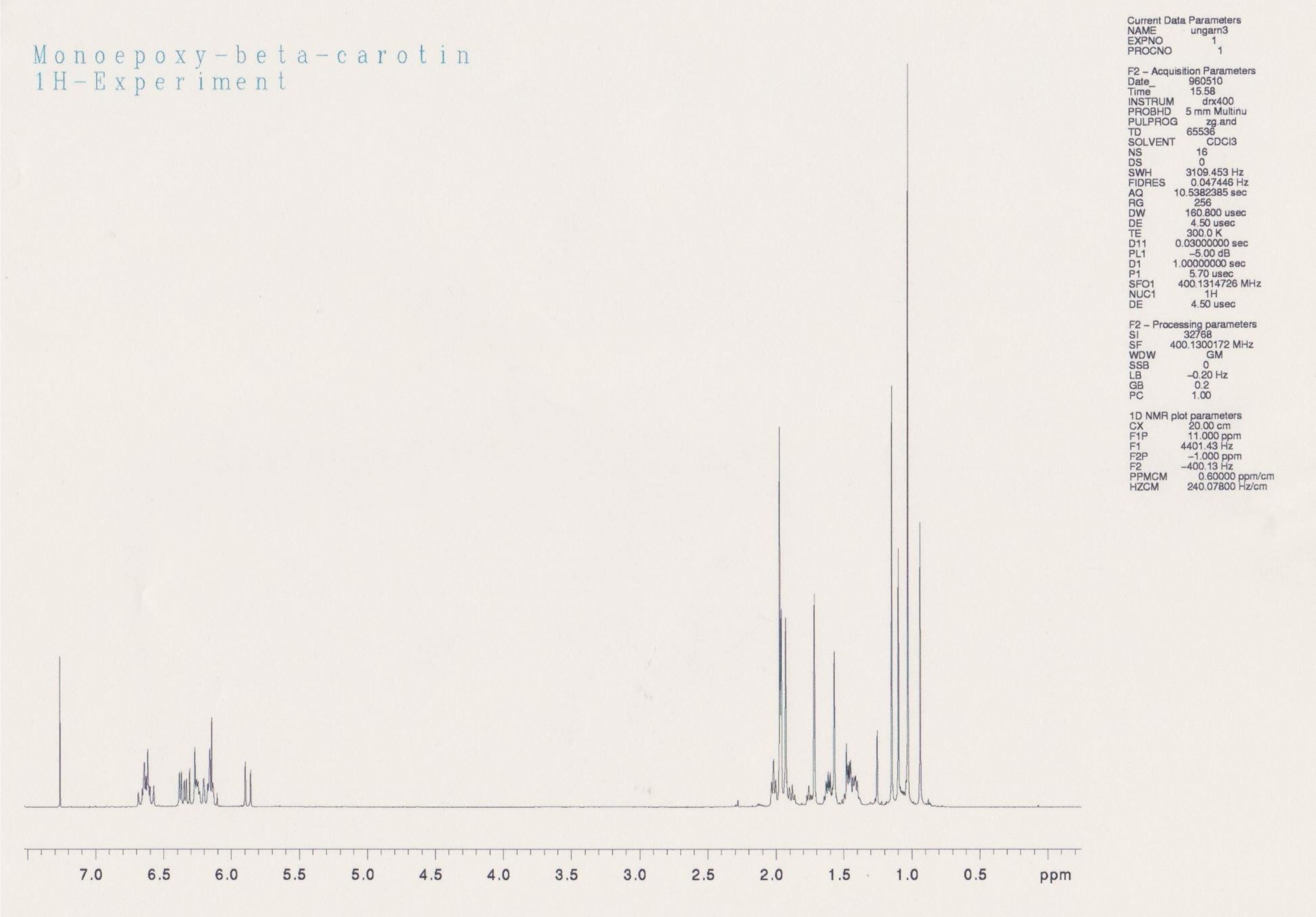

Figure S2.b. ^{1}H-NMR spectrum of β-carotene 5,6-epoxide (**10**) in $CDCl_3$ (400 MHz) (5.8-6.7 ppm)

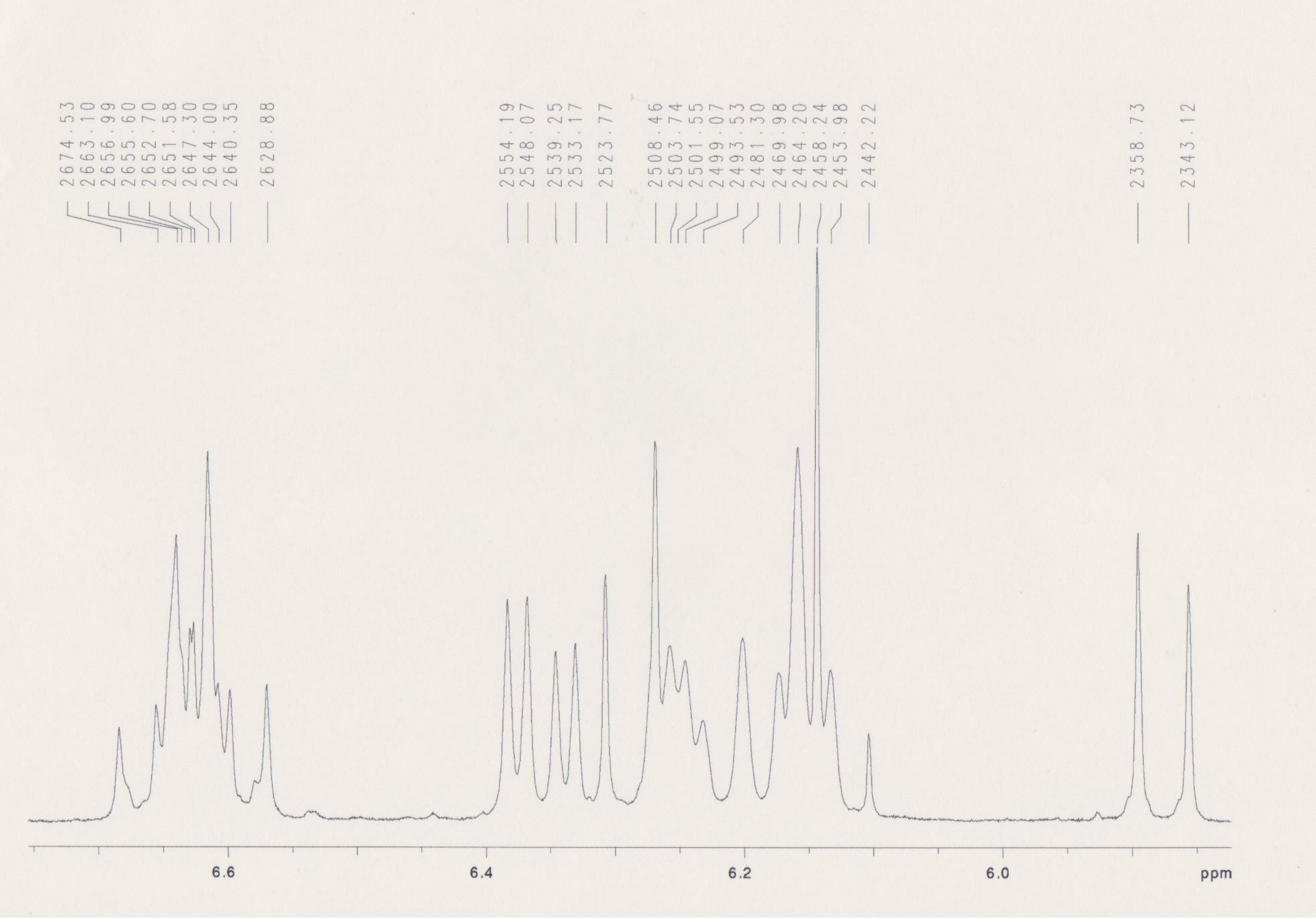

Figure S2.c. ^{1}H-NMR spectrum of β-carotene 5,6-epoxide (**10**) in $CDCl_3$ (400 MHz) (0.9-2.1 ppm)

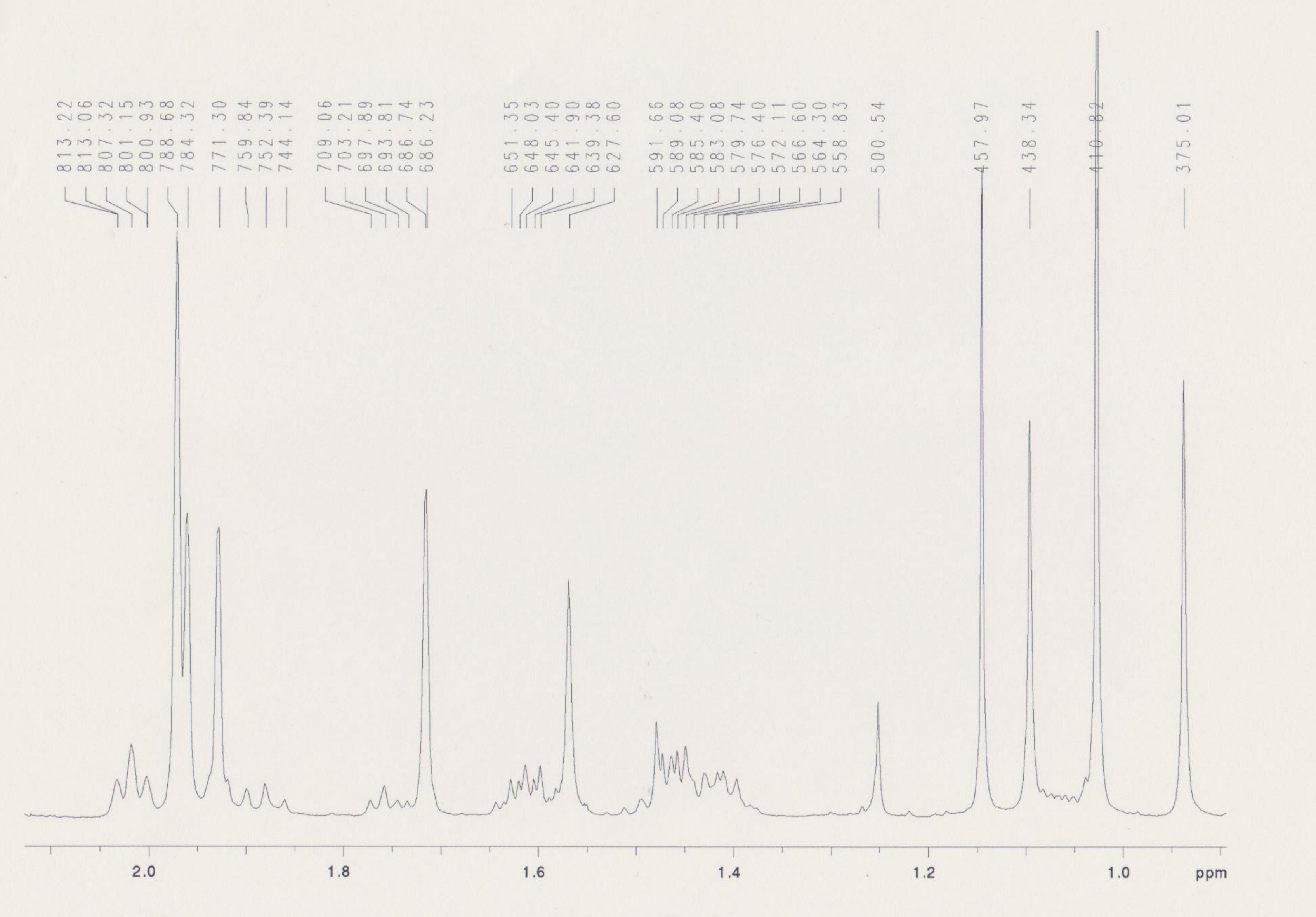

Figure S3.a. $^{1}H,^{1}H$-COSY NMR spectrum of β-carotene 5,6-epoxide (**10**) in $CDCl_3$ (400 MHz)

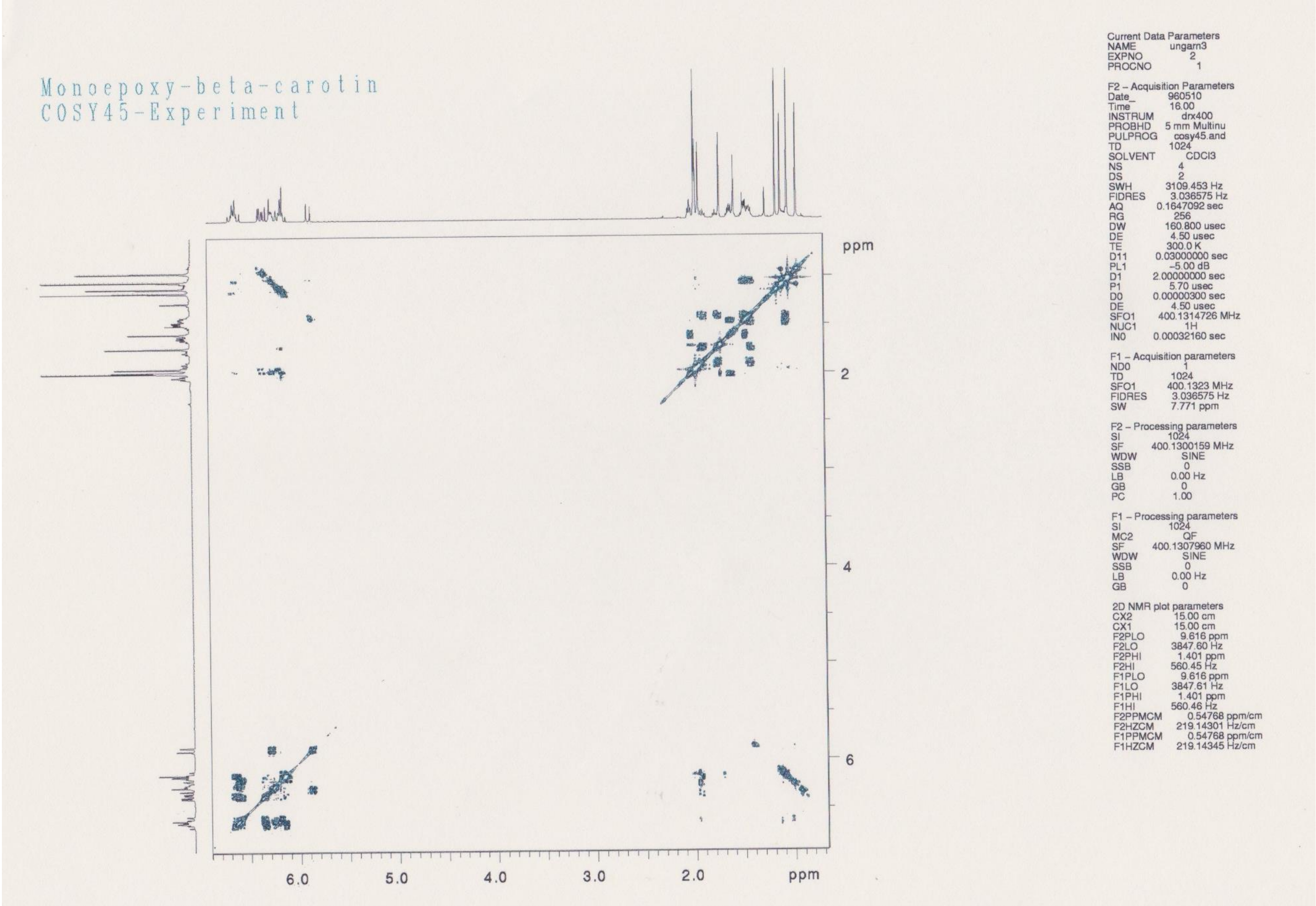

Figure S3.b. $^{1}H,^{1}H$-COSY NMR spectrum of β-carotene 5,6-epoxide (**10**) in $CDCl_3$ (400 MHz) (0-2.1 ppm)

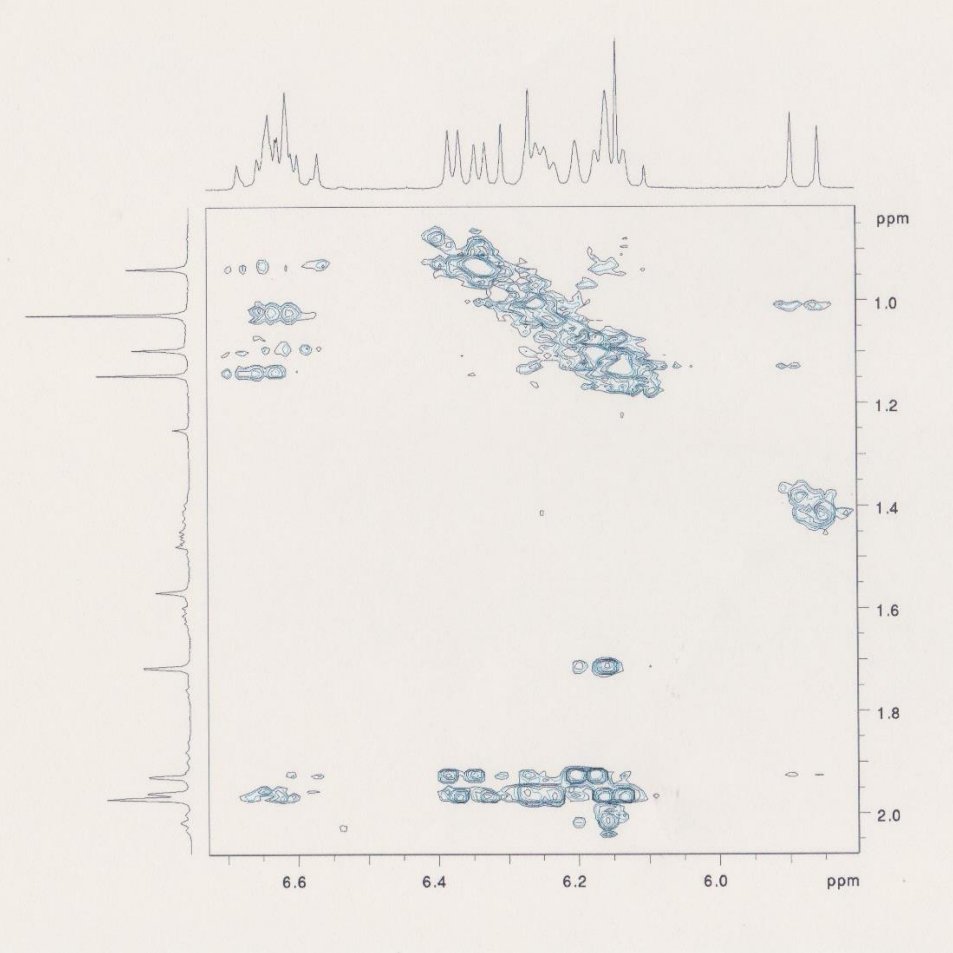

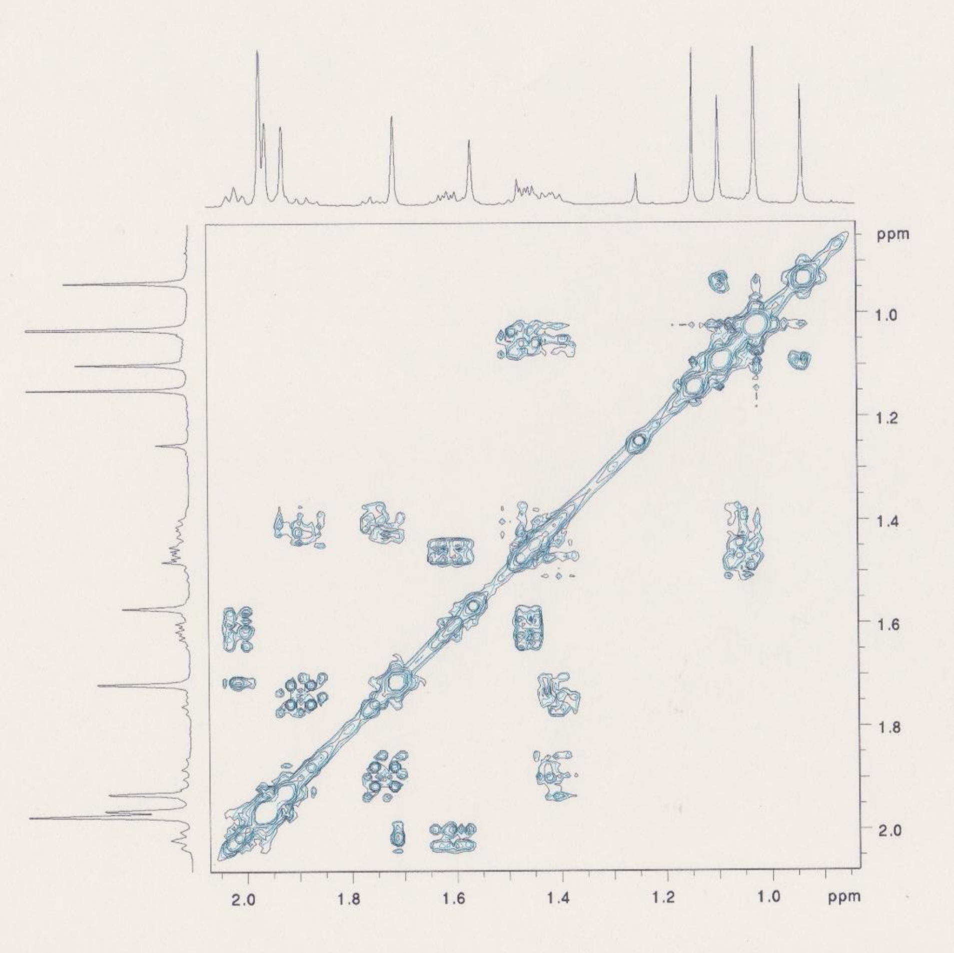

Figure S3.c. $^1H,^1H$-COSY NMR spectrum of β-carotene 5,6-epoxide (**10**) in $CDCl_3$ (400 MHz) (5.8-6.7 ppm)

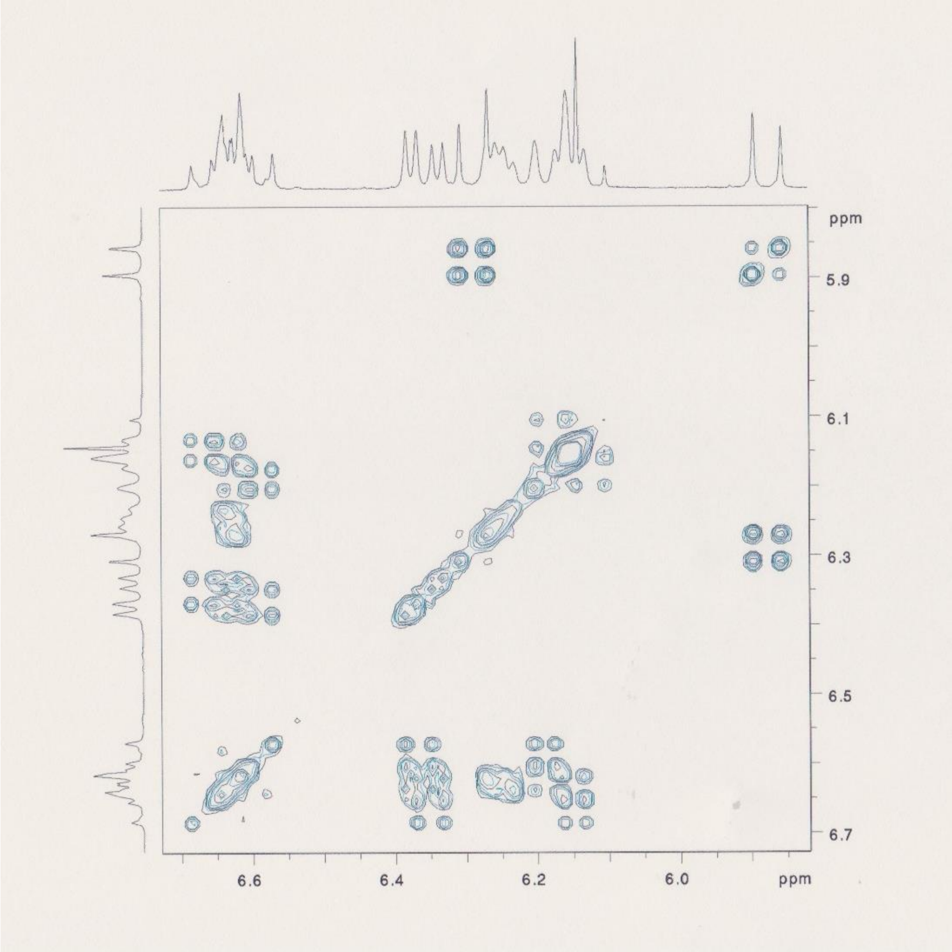

Figure S4.a. ^{13}C-NMR spectrum of β-carotene 5,6-epoxide (**10**) in $CDCl_3$

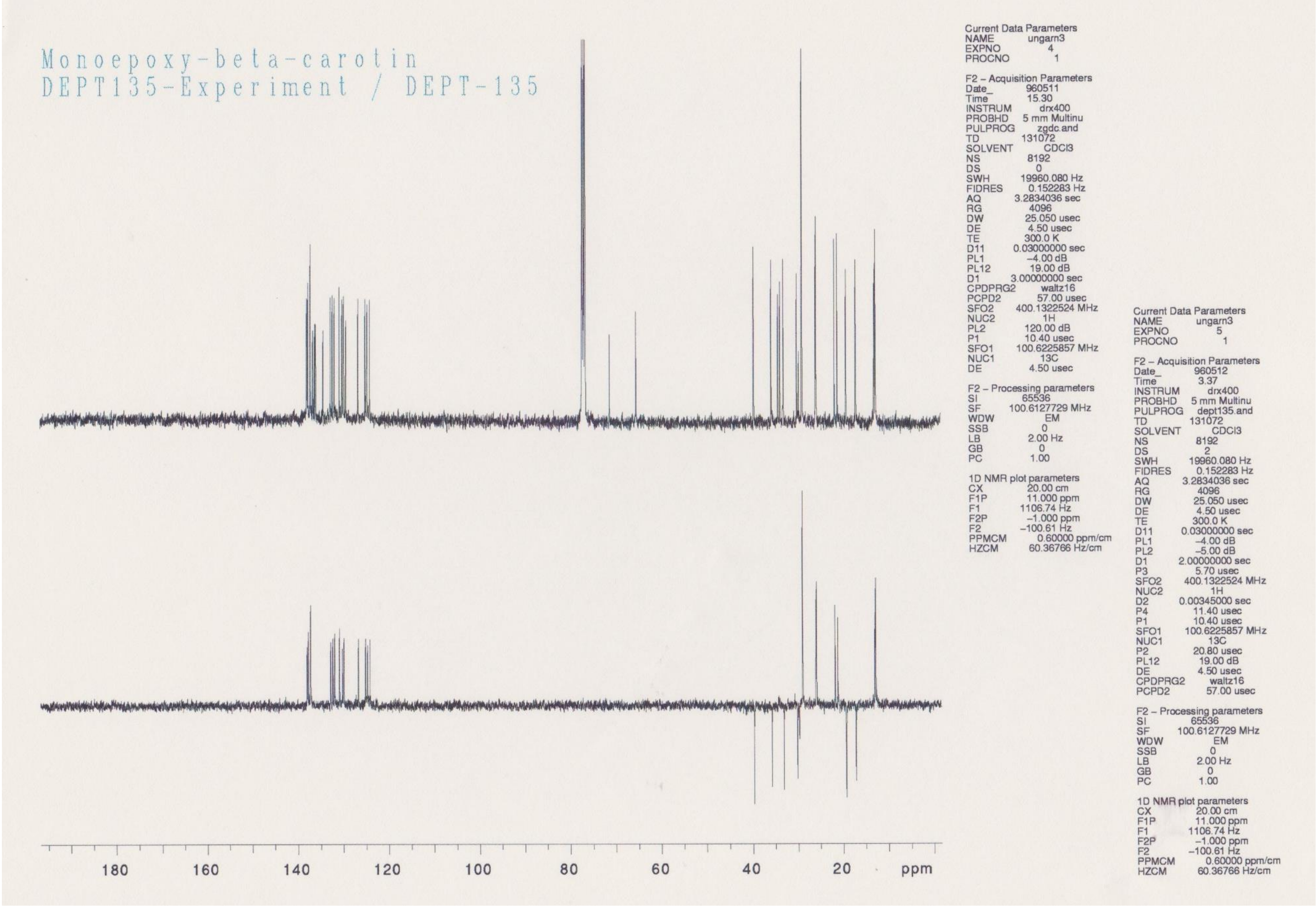

Figure S4.b. ^{13}C-NMR spectrum of β-carotene 5,6-epoxide (**10**) in $CDCl_3$ (124-138 ppm)

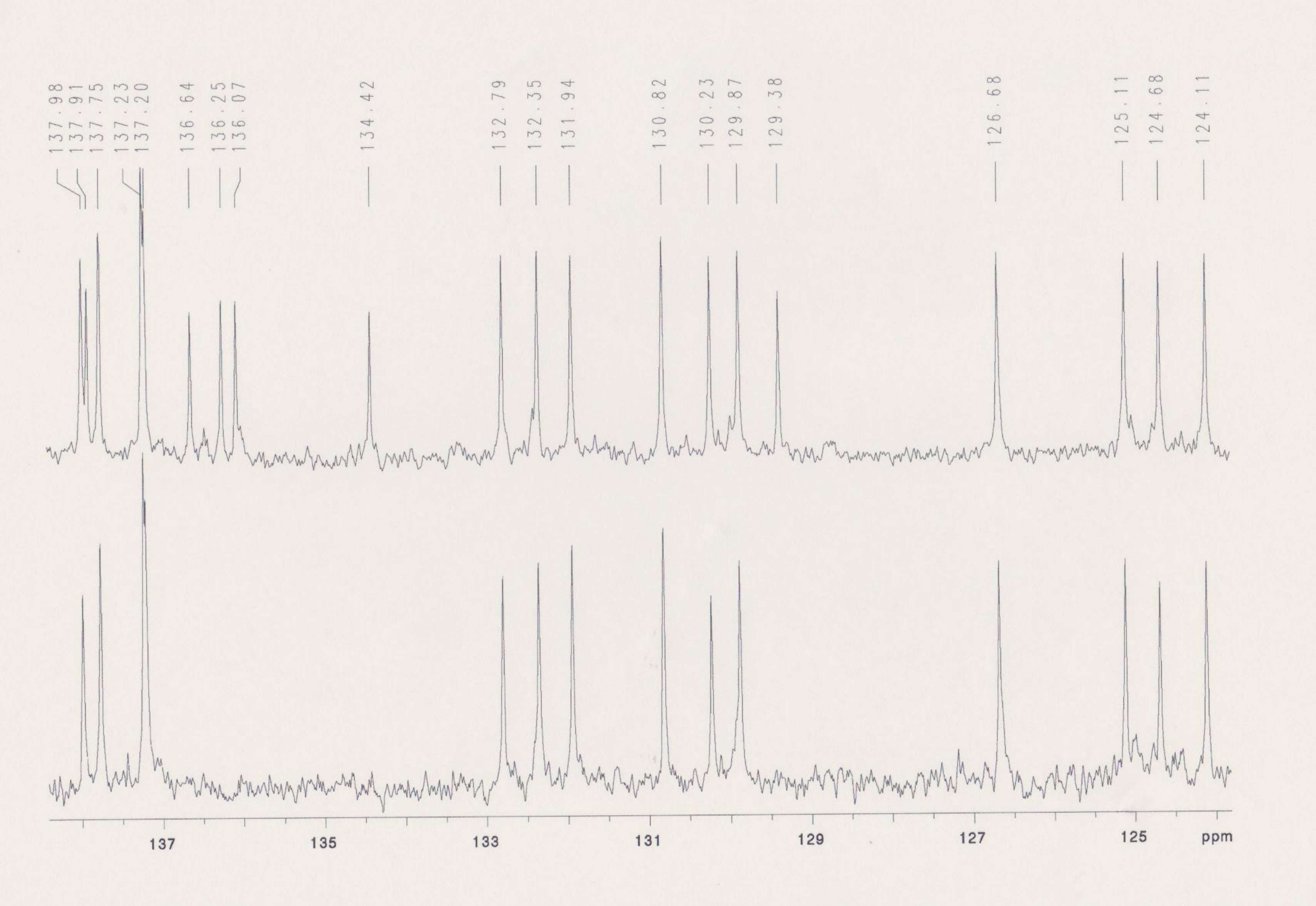

Figure S4.c. ^{13}C-NMR spectrum of β-carotene 5,6-epoxide (**10**) in $CDCl_3$ (10-72 ppm)

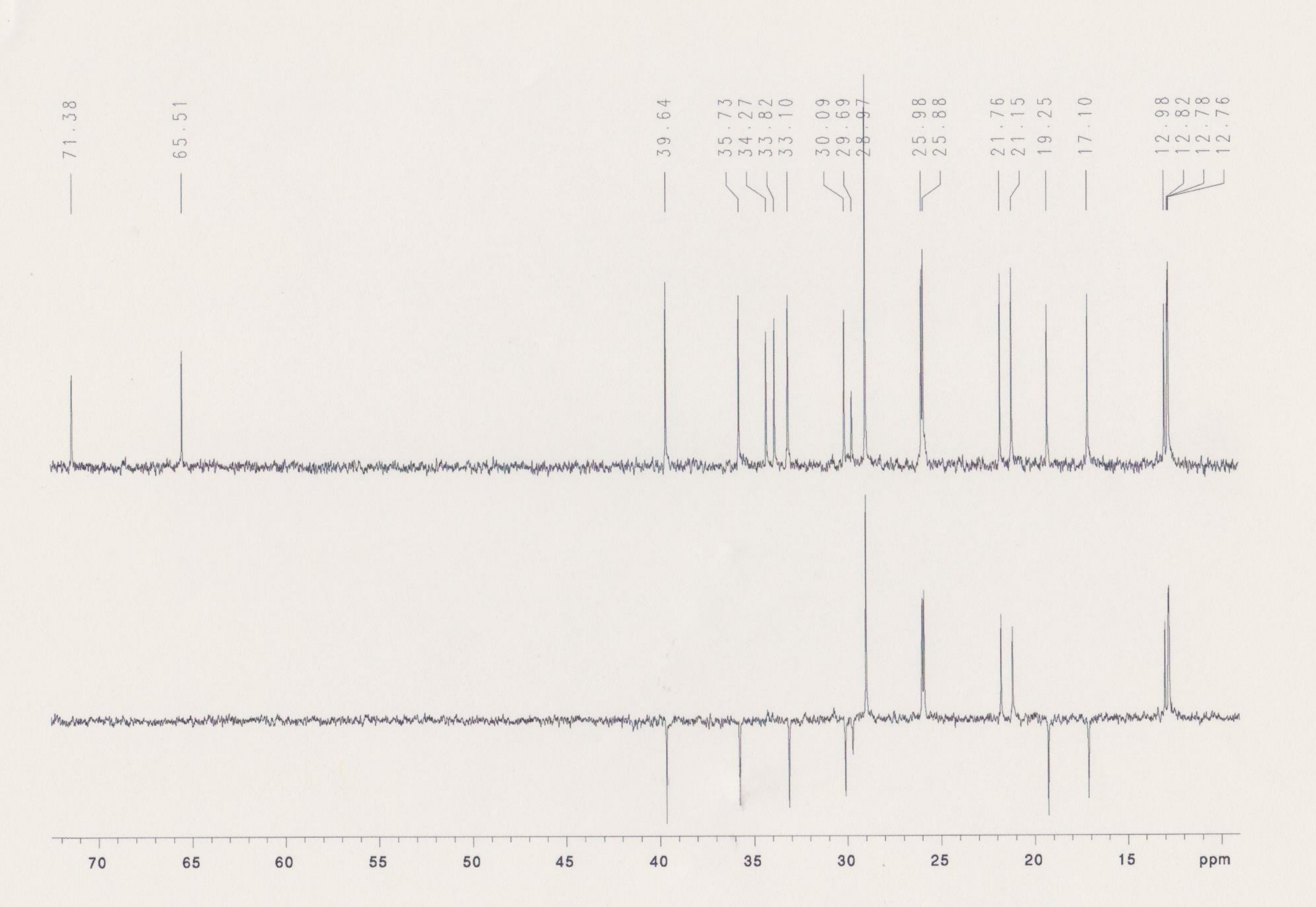

Figure S5.a ^{1}H,^{13}C-HMQC NMR spectrum of β-carotene 5,6-epoxide (**10**) in $CDCl_3$

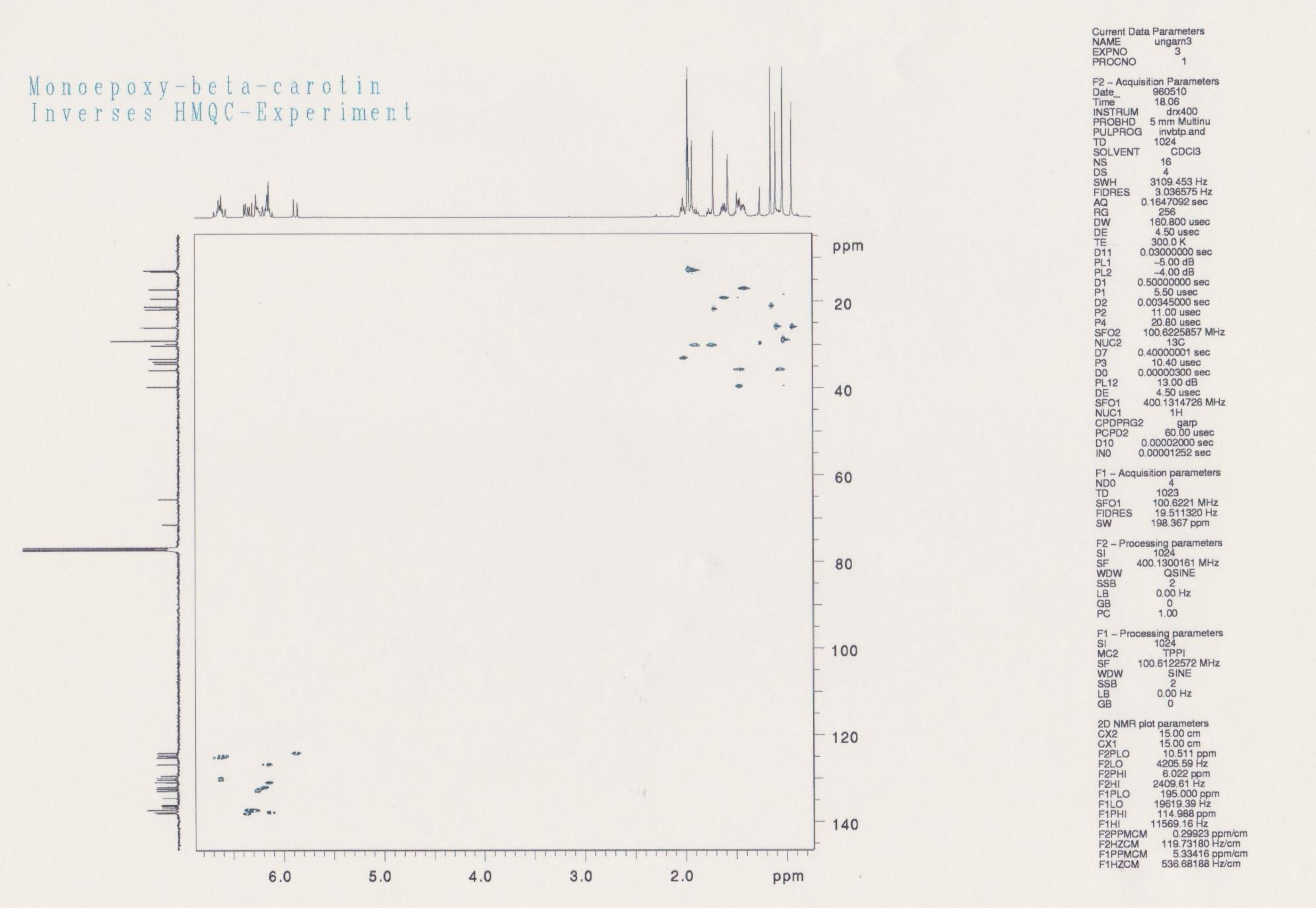

Figure S5.b $^{1}H,^{13}C$-HMQC NMR spectrum of β-carotene 5,6-epoxide (**10**) in $CDCl_3$ (10-72 ppm)

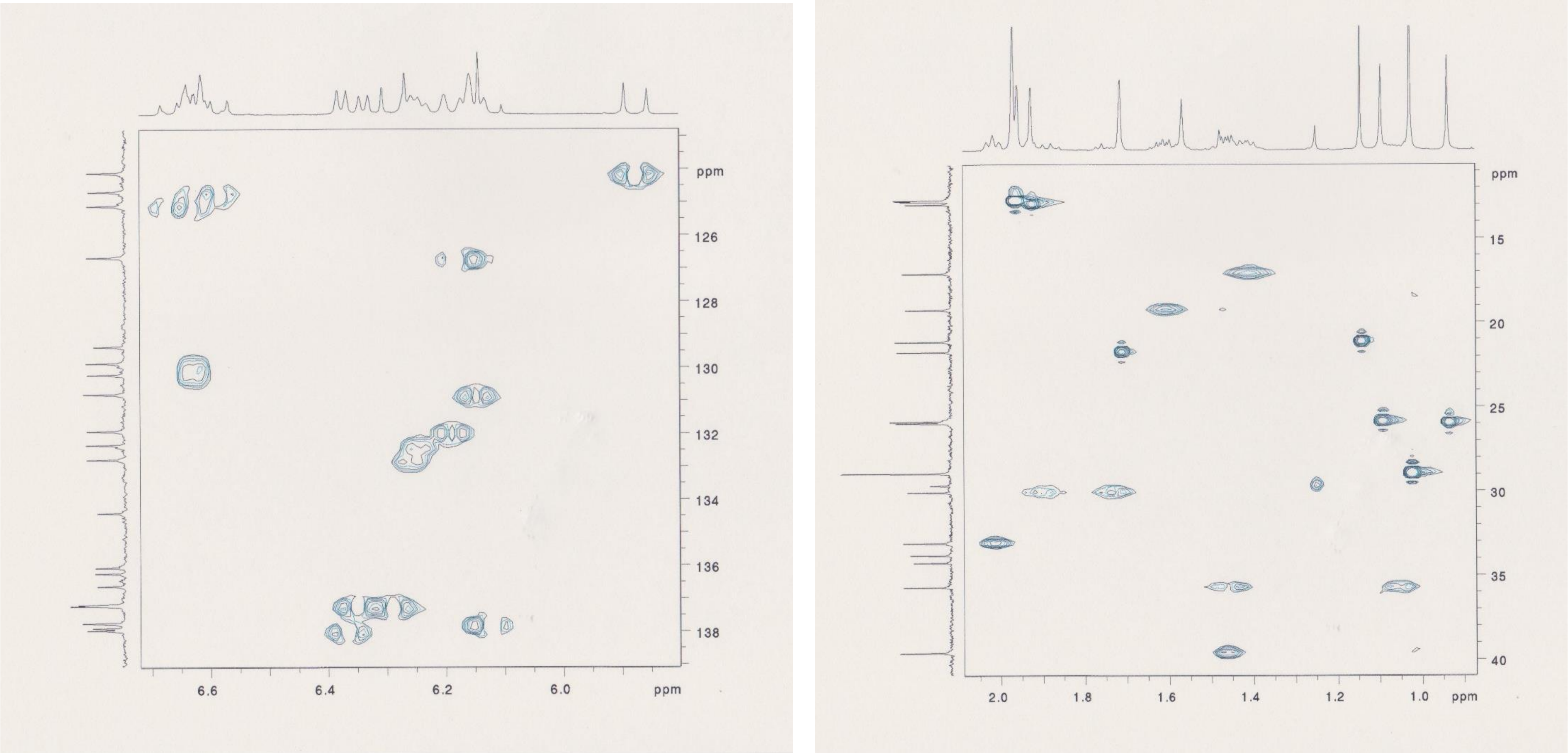

Figure S6.a. ^{1}H-NMR spectrum of β-carotene 5,6,5′,6′-diepoxide (**11**) in $CDCl_3$ (400 MHz)

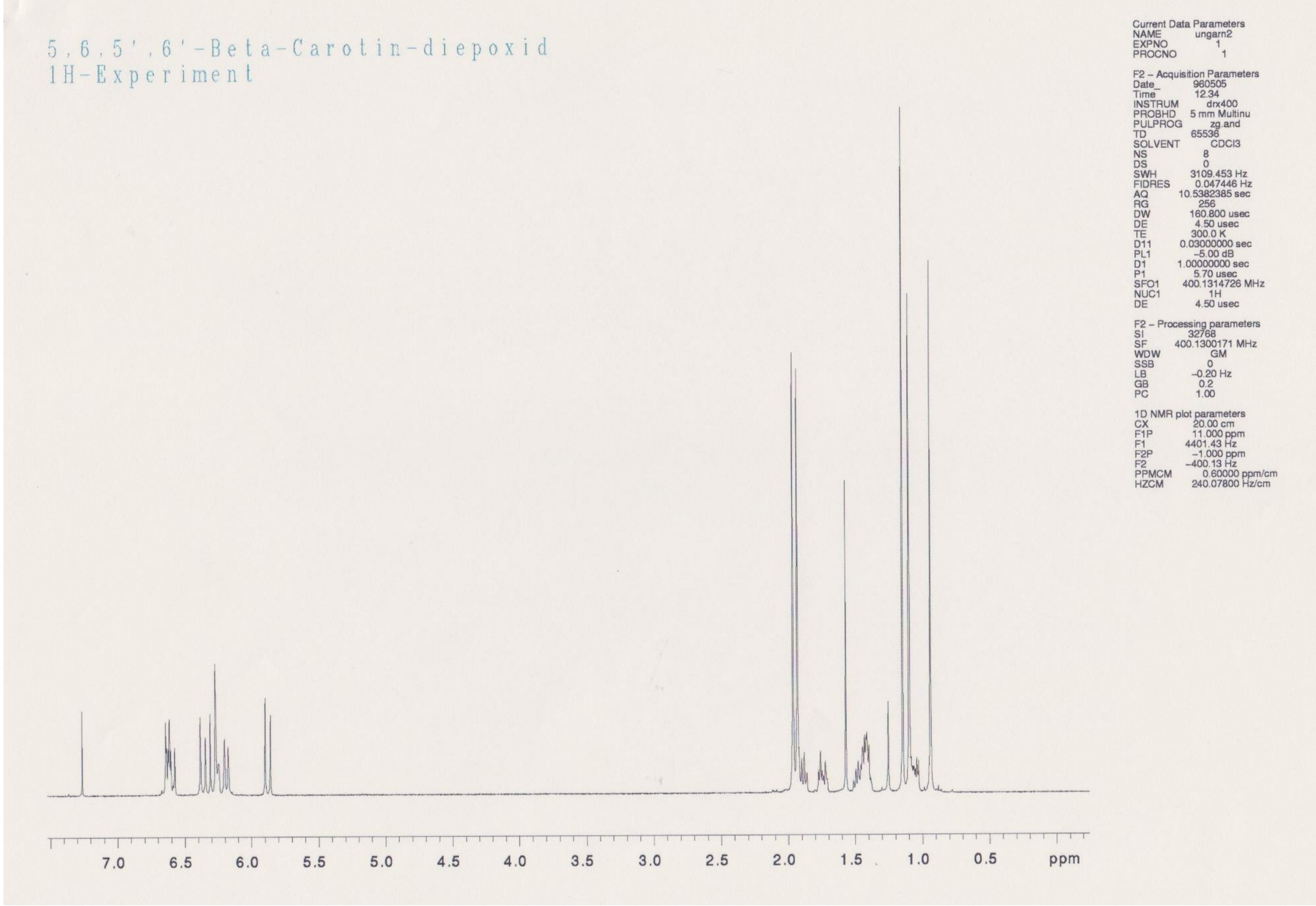

Figure S6.b. ^{1}H-NMR spectrum of β-carotene 5,6,5′,6′-diepoxide (**11**) in $CDCl_3$ (400 MHz) (5.8-6.7 ppm)

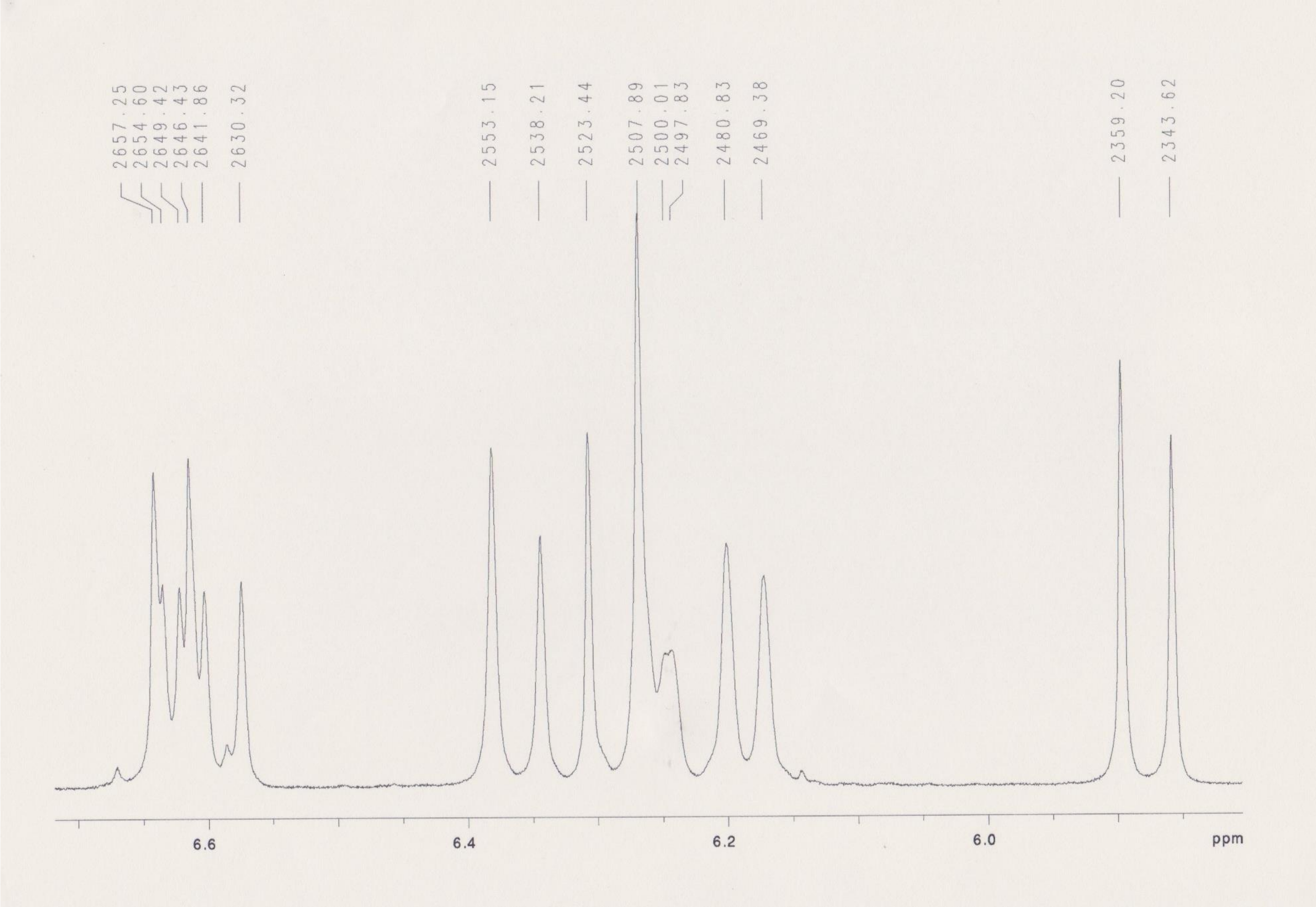

Figure S6.c. ^{1}H-NMR spectrum of β-carotene 5,6,5′,6′-diepoxide (**11**) in $CDCl_3$ (400 MHz) (0.9-2.0 ppm)

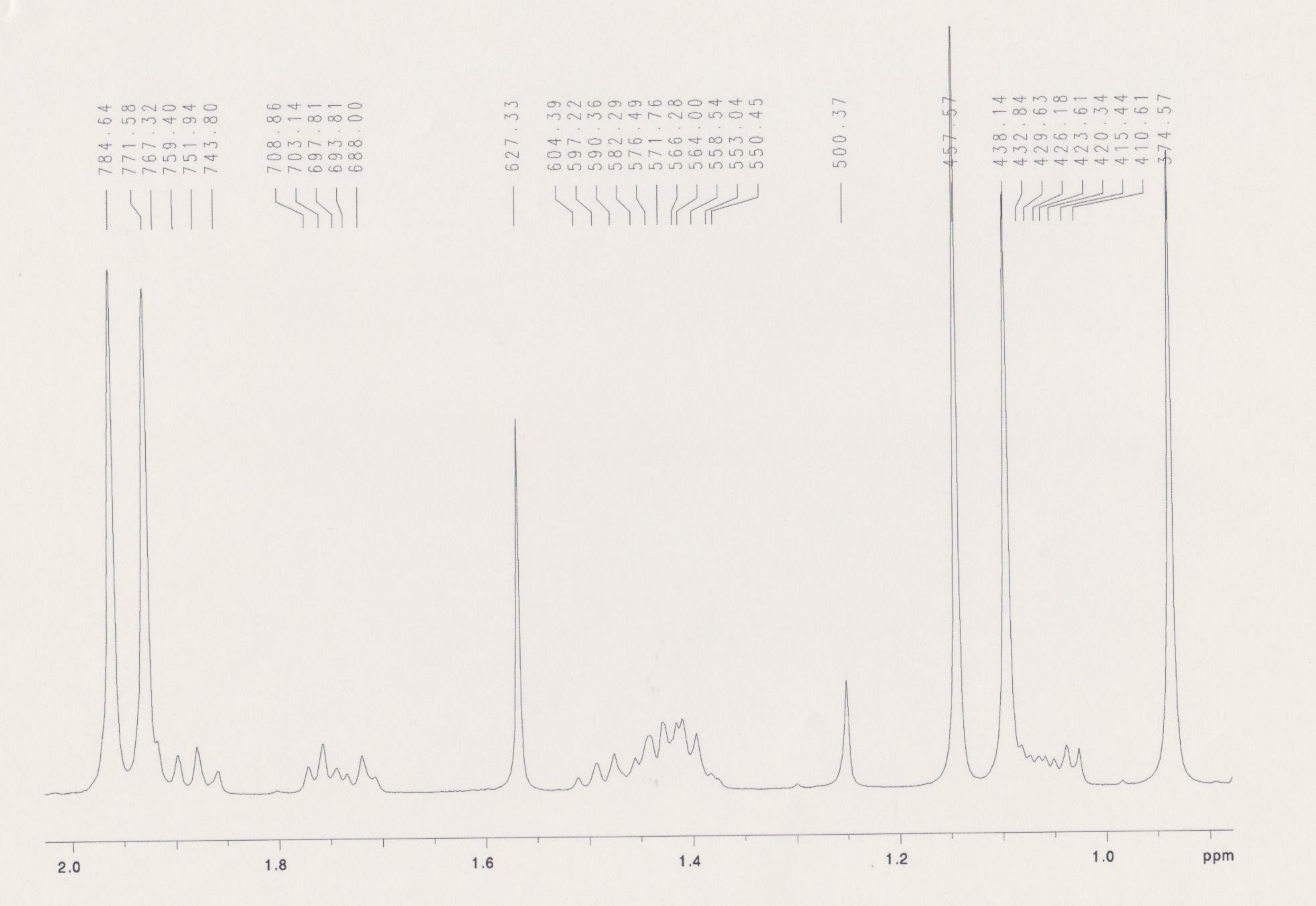

Figure S7.a. $^1H,^1H$-COSY NMR spectrum of β-carotene 5,6,5′,6′-diepoxide (**11**) in $CDCl_3$ (400 MHz)

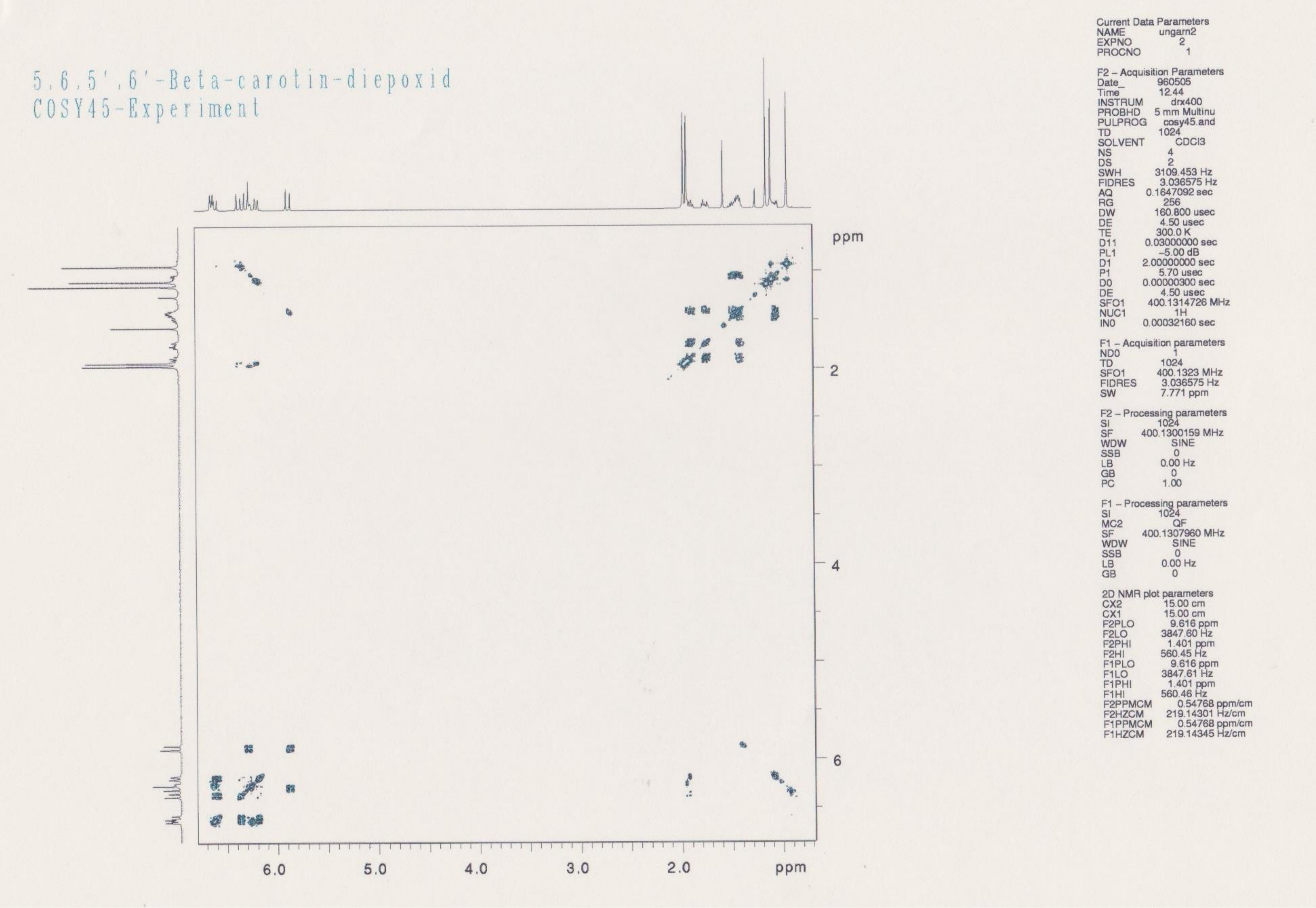

Figure S7.b. $^{1}H,^{1}H$-COSY NMR spectrum of β-carotene 5,6,5′,6′-diepoxide (**11**) in $CDCl_3$ (400 MHz) (0.9-2.0 ppm)

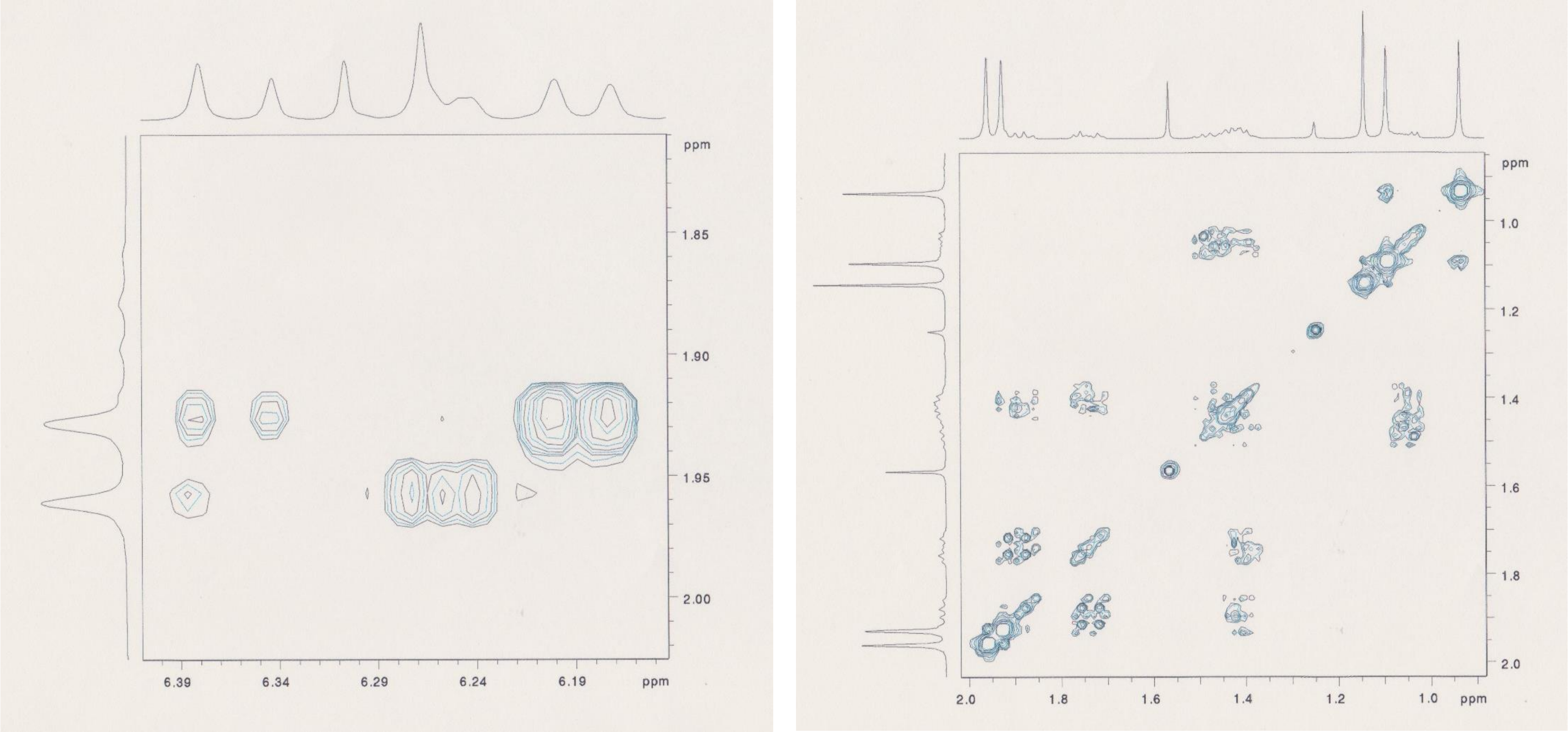

Figure S7.c. ¹H,¹H-COSY NMR spectrum of β-carotene 5,6,5′,6′-diepoxide (**11**) in $CDCl_3$ (400 MHz) (5.7-6.7 ppm)

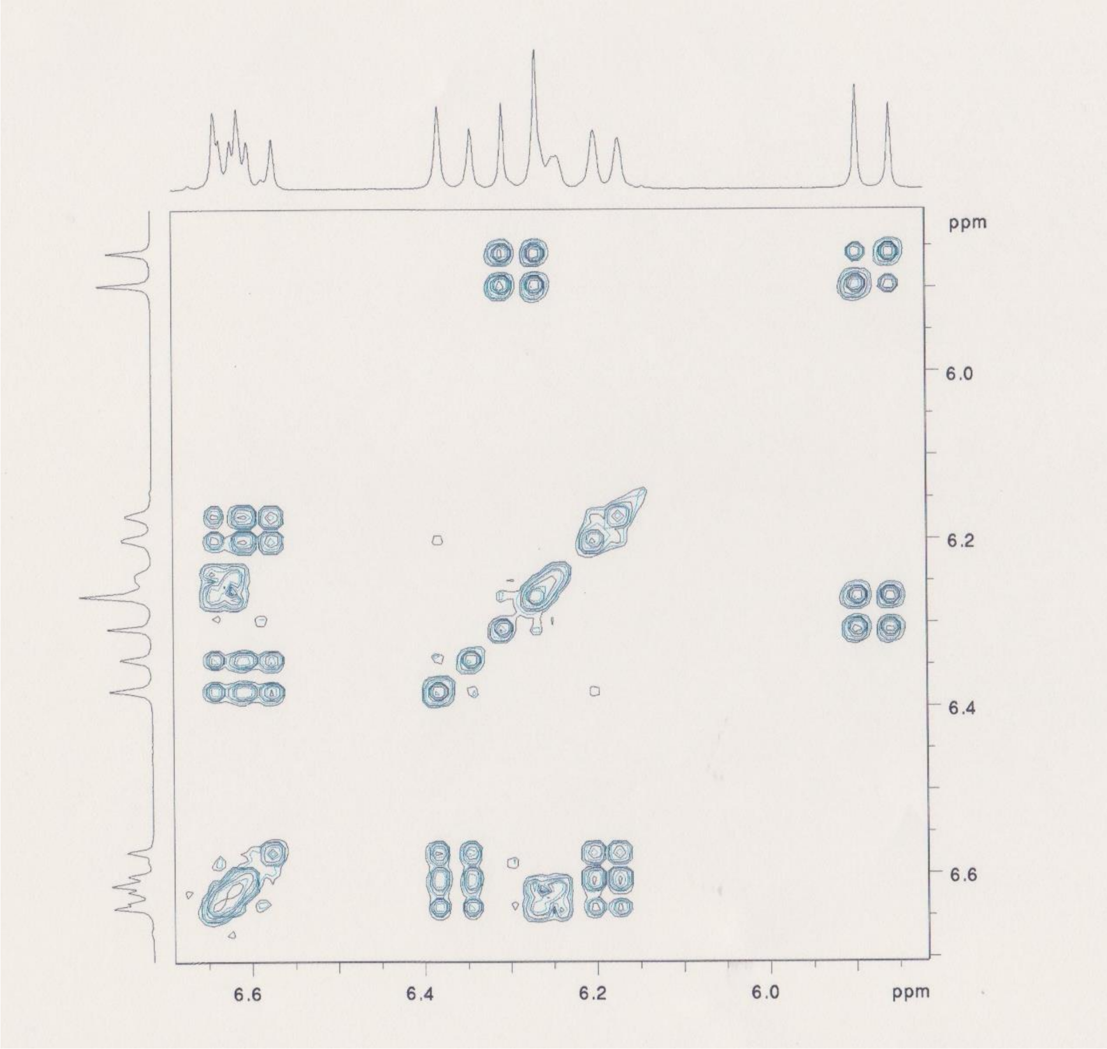

Figure S8. ^{13}C-NMR spectrum of β-carotene 5,6,5′,6′-diepoxide (**11**) in $CDCl_3$

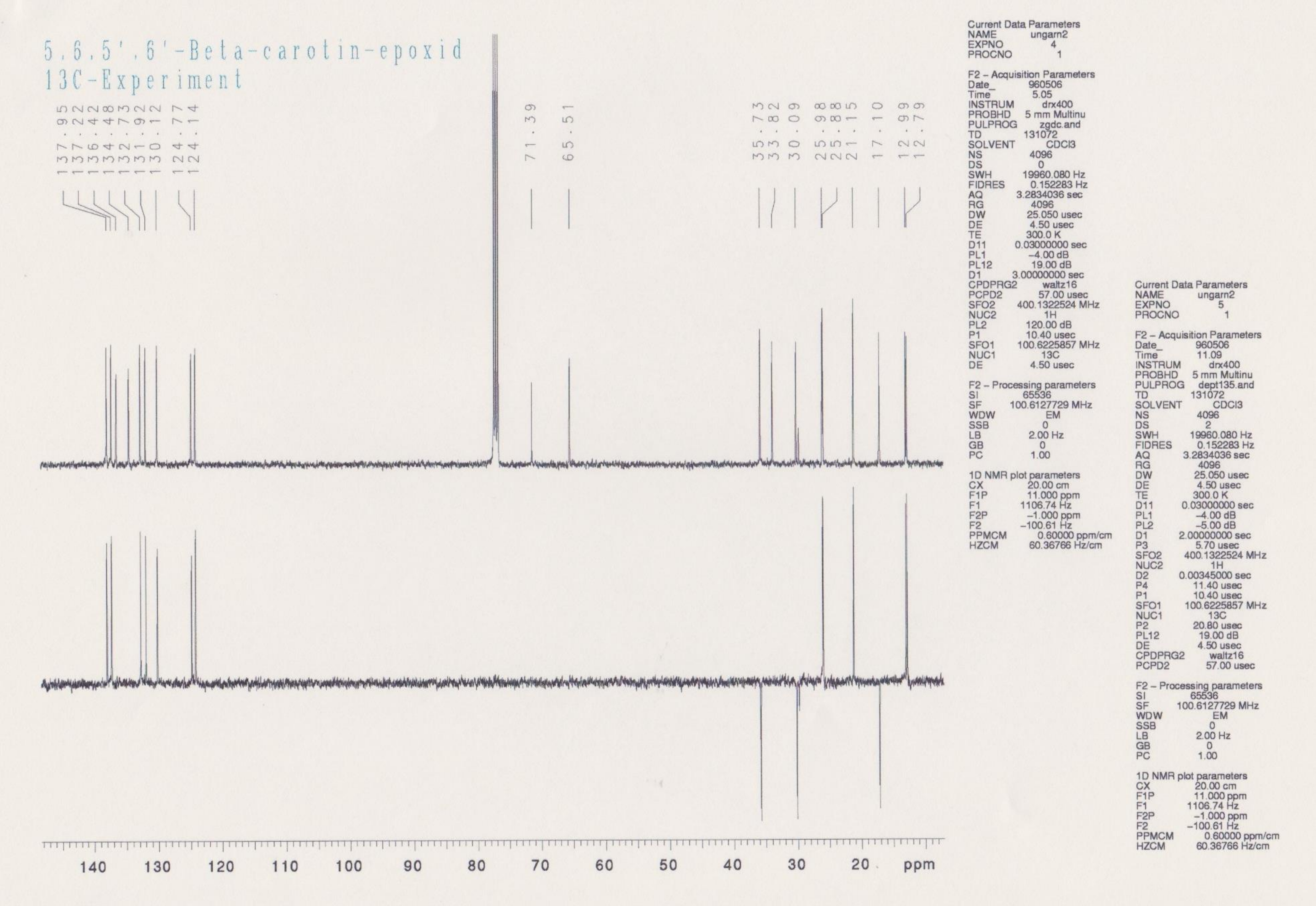

Figure S9.a. ^{1}H,^{13}C-HMQC NMR spectrum of β-carotene 5,6,5′,6′-diepoxide (**11**) in $CDCl_3$

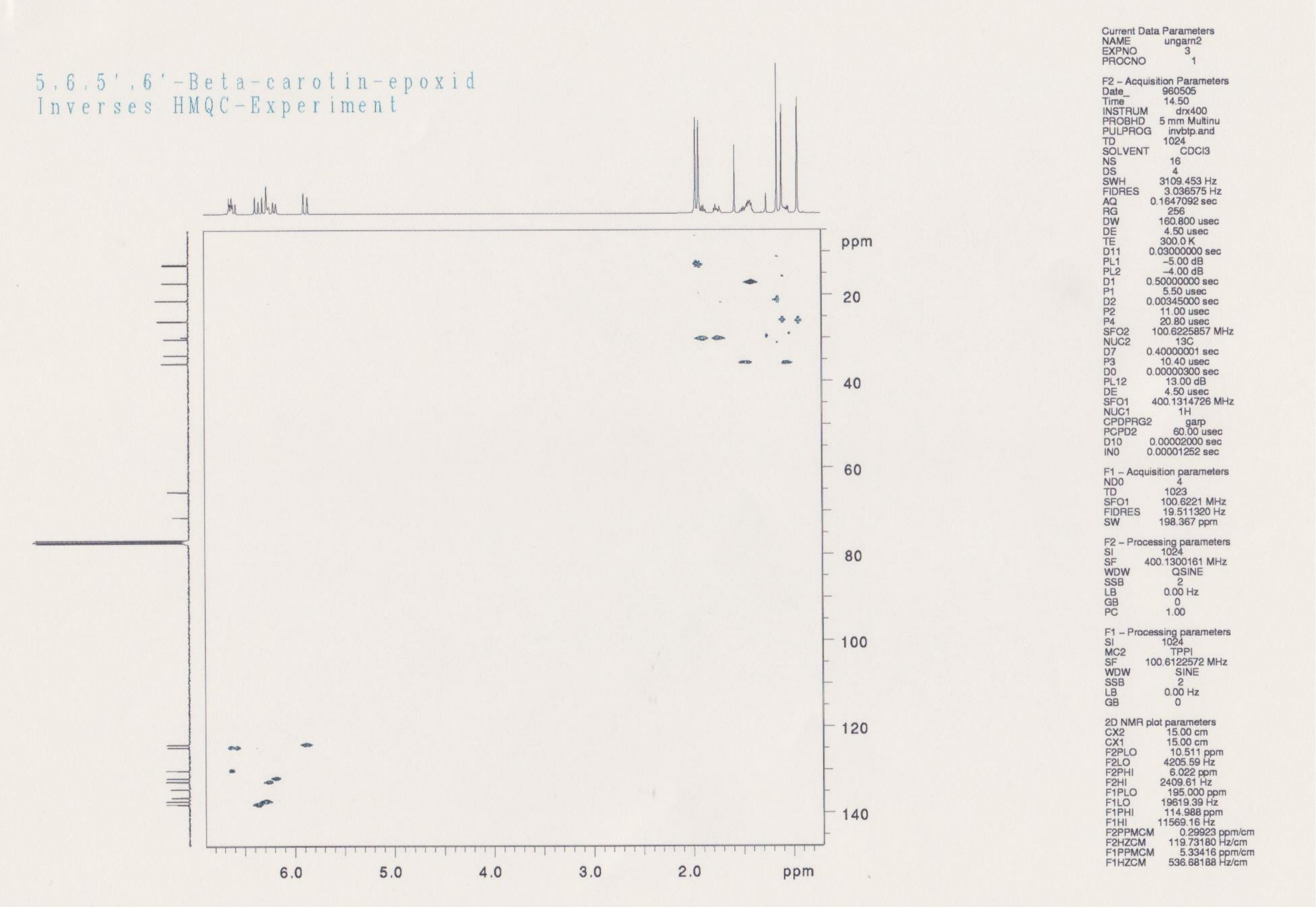

Figure S9.b. $^{1}H,^{13}C$-HMQC NMR spectrum of β-carotene 5,6,5′,6′-diepoxide (**11**) in $CDCl_3$

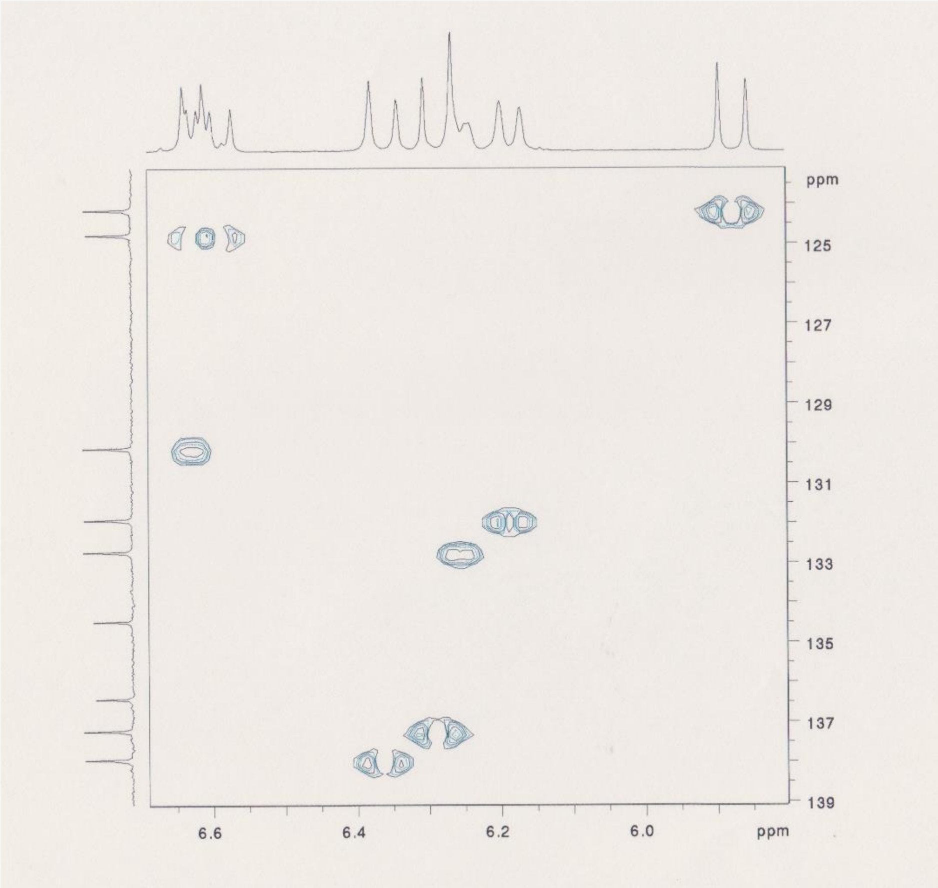

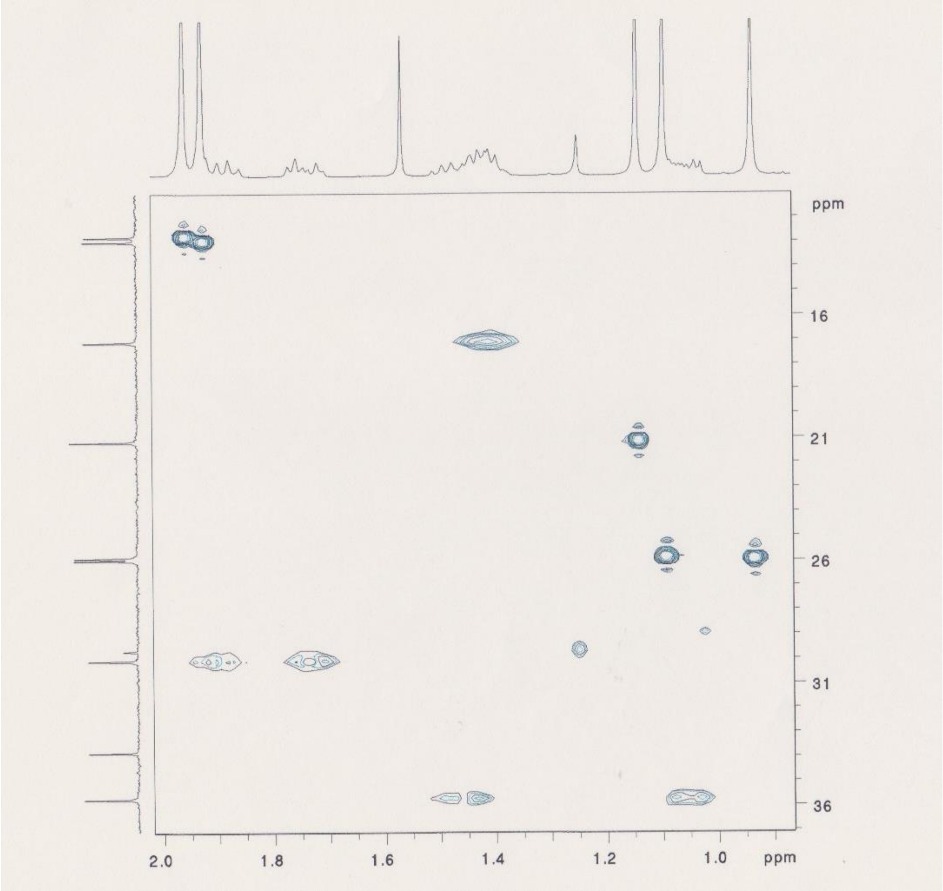

Figure S10. UV-vis and EIC chromatogram of *Teleki speciosa* flower extract.

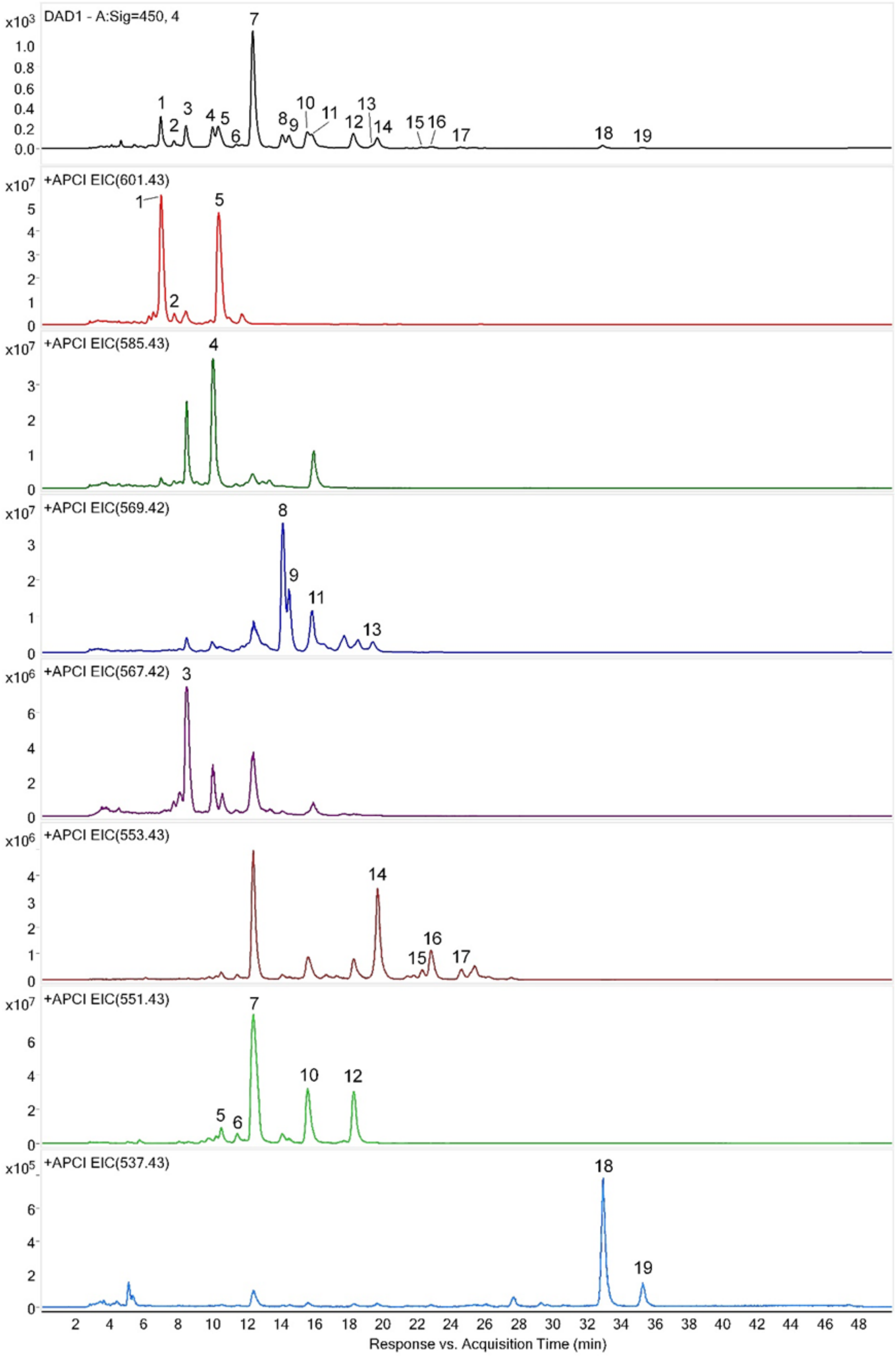

Figure S11. UV-vis spectra of catotenoids in *Telekia speciosa* flower extract detected by HPLC-DAD

Peak 1: Violaxanthin

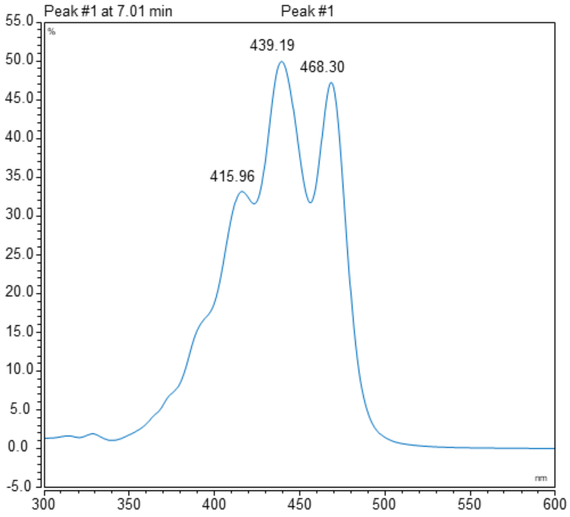

Peak 2: (9Z)-Neoxanthin

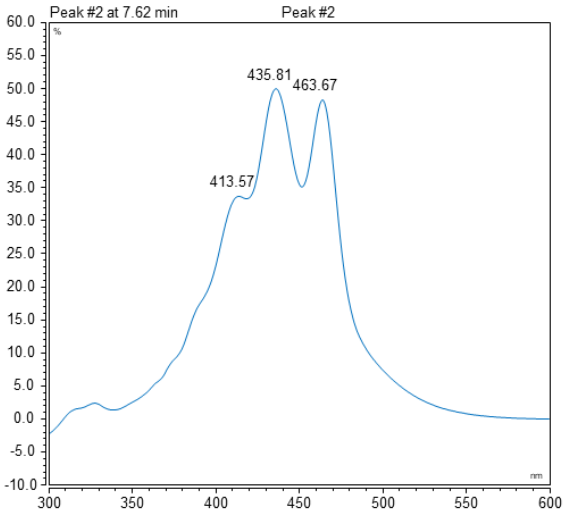

Peak 3: Lutein 5,6-epoxide

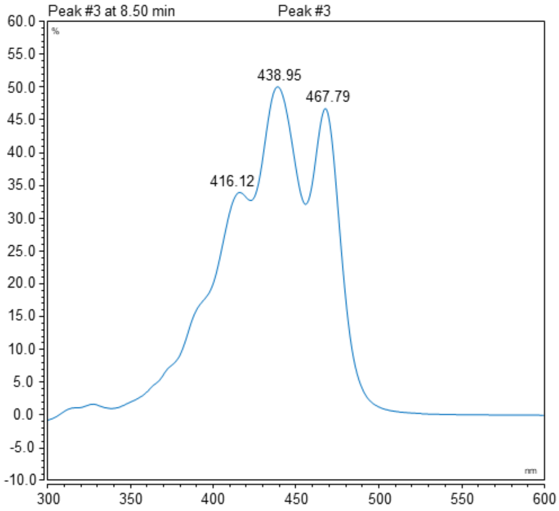

Peak 4: Antheraxanthin

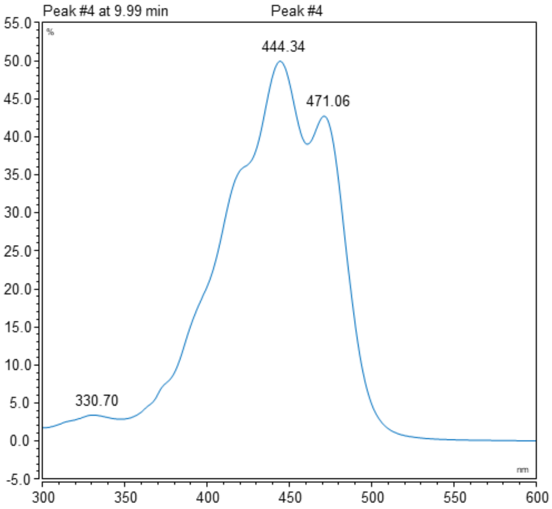

Peak 5: (9Z)-Violaxanthin

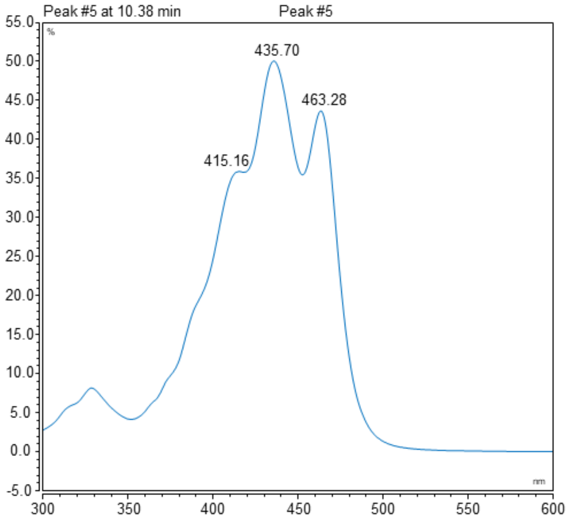

Peak 6: (13′Z)-Lutein

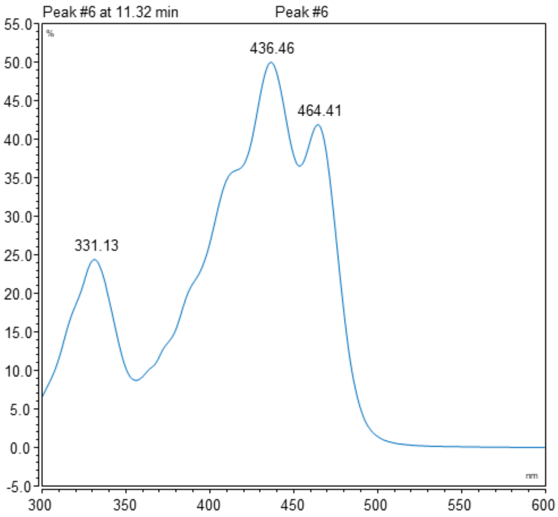

Peak 7: Lutein

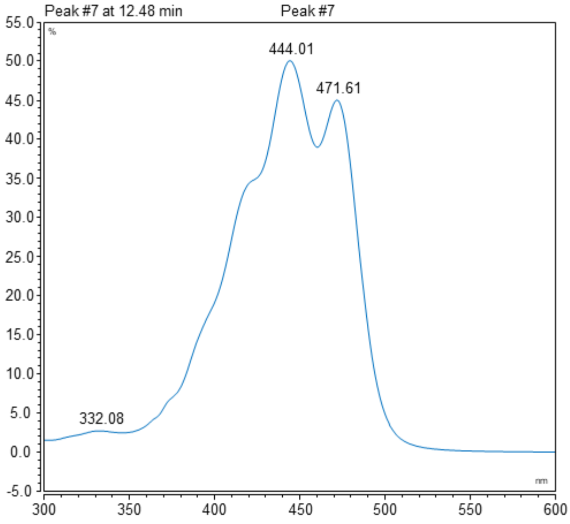

Peak 8: β-Carotene diepoxide

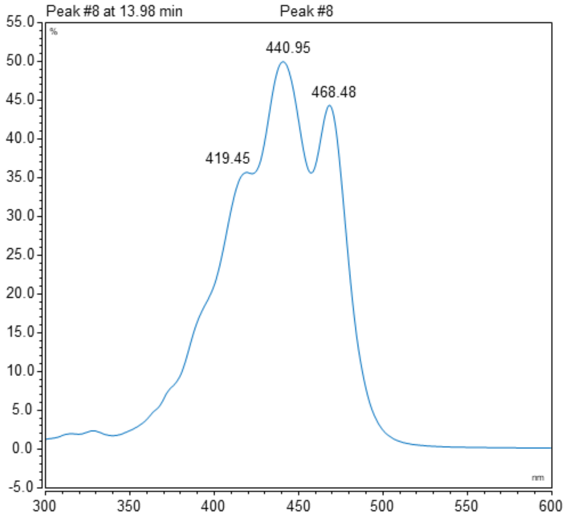

Peak 9: Zeaxanthin

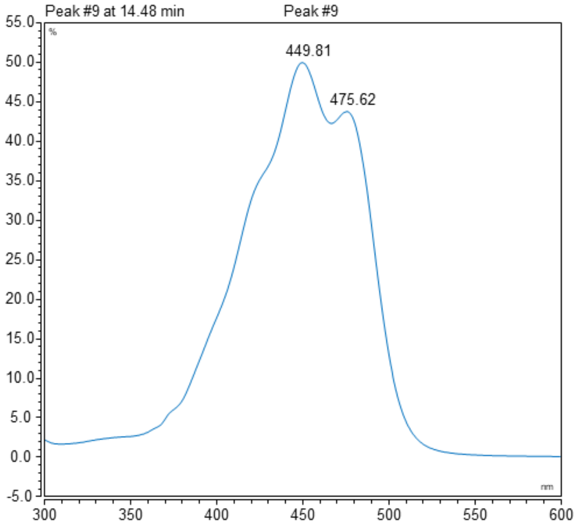

Peak 10: (9Z)-Lutein

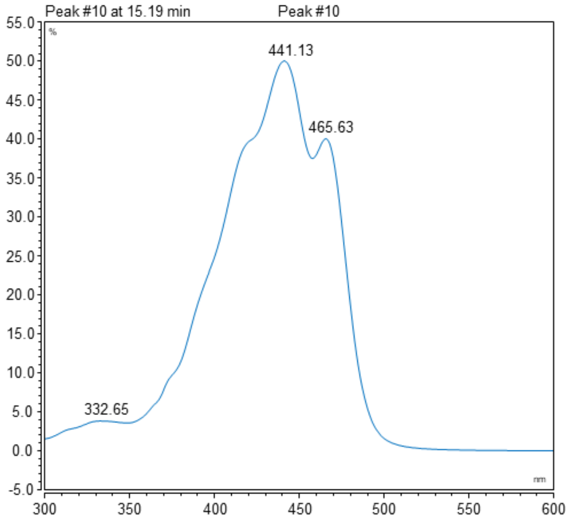

Peak 11: Aurochrome

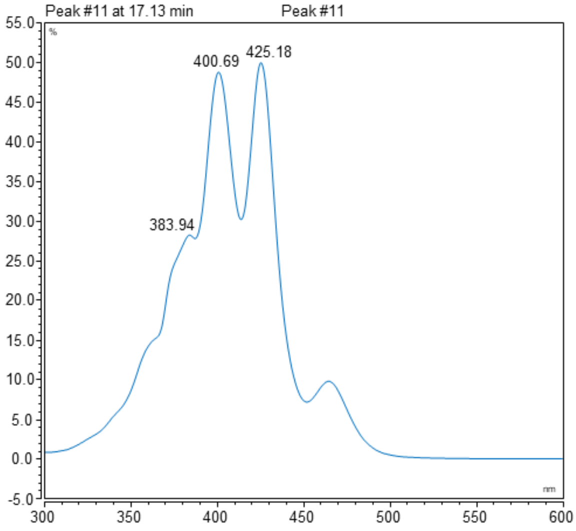

Peak 12: (9′Z)-Lutein

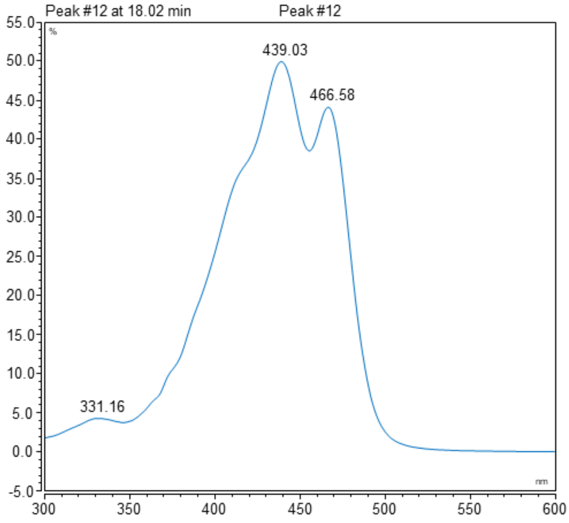

Peak 13: (9*Z*)-Zeaxanthin

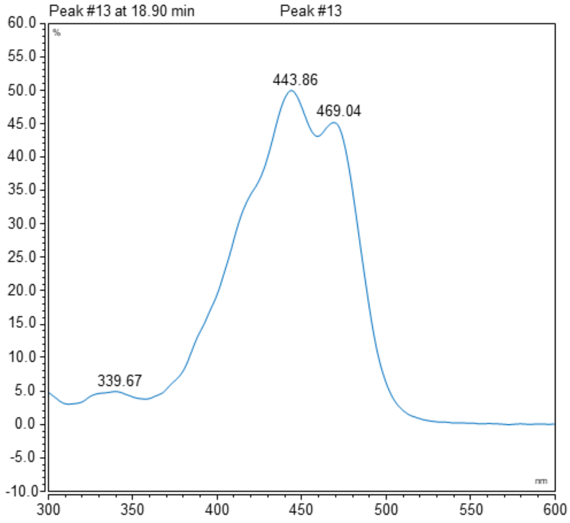

Peak 14: *α*-Cryptoxanthin

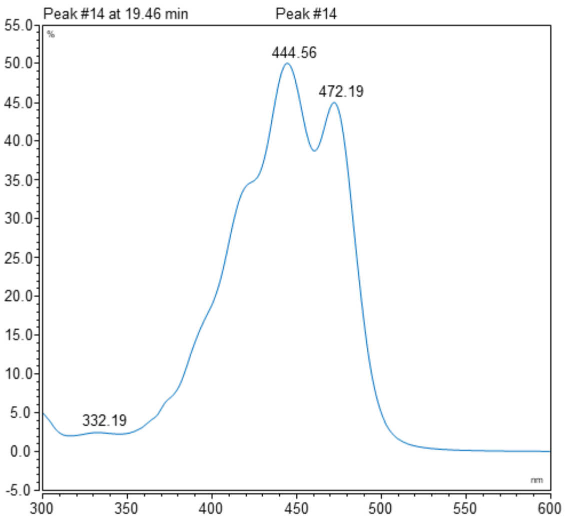

Peak 15: (9*Z*)-*α*-Cryptoxanthin

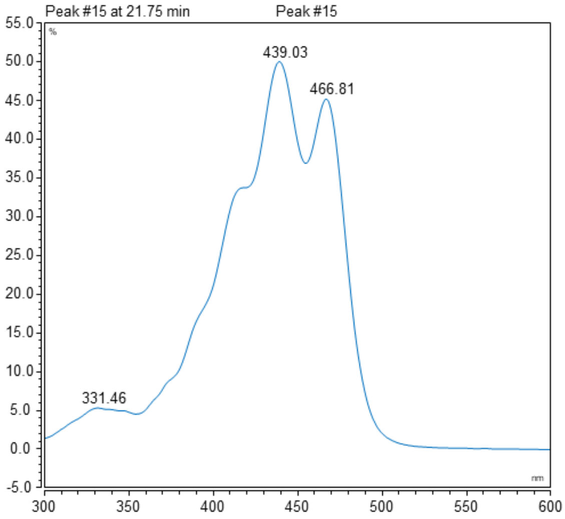

Peak 16: β-Carotene 5,6-epoxide

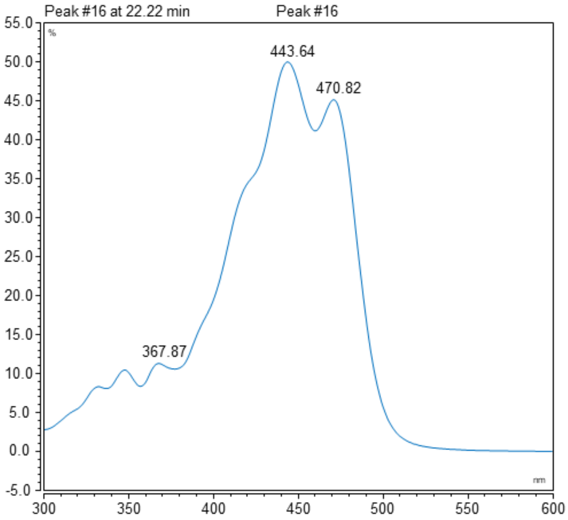

Peak 17: β-Carotene 5,8-epoxide

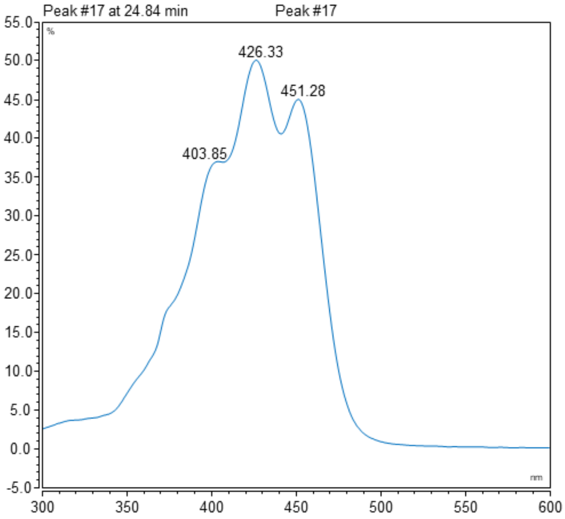

Peak 18: β-Carotene

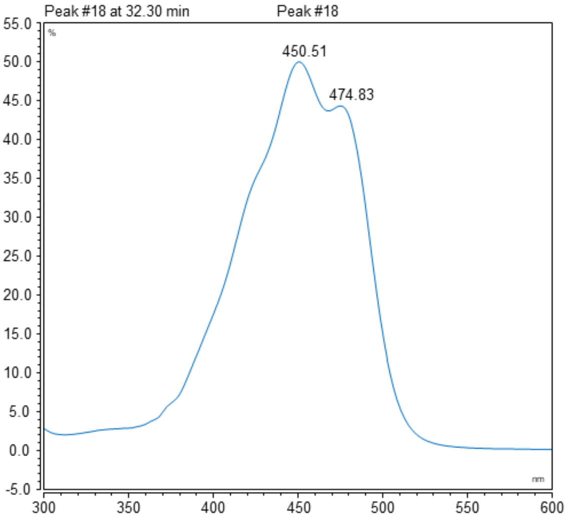

Peak 19: (9*Z*)-β-Carotene

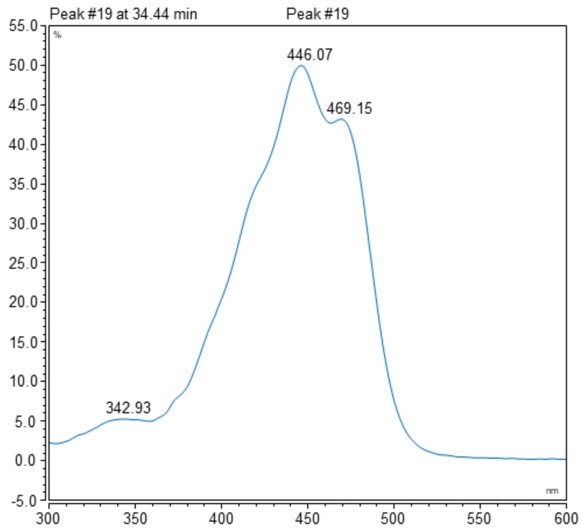

Supplement: Supplementary file 1 [file plants-12-04116-s001.zip › plants-2737711-supplementary.pdf]
